# A deep intronic mutation causes RAD50 deficiency through an unusual mechanism of distant exon activation

**DOI:** 10.1172/JCI178528

**Published:** 2024-12-12

**Authors:** Kristine Bousset, Stefano Donega, Najim Ameziane, Tabea Fleischhammer, Dhanya Ramachandran, Miriam Poley-Gil, Detlev Schindler, Ingrid M. van de Laar, Franco Pagani, Thilo Dörk

**Affiliations:** 1 Gynecology Research Unit, Hannover Medical School, Hannover, Germany.; 2 International Centre for Genetic Engineering and Biotechnology, Trieste, Italy, and Intramural Research Program of National Institute on Aging, NIH, Baltimore, Maryland, USA.; 3 UMC Cancer Center, Amsterdam, Netherlands.; 4 Institute of Human Genetics, University of Würzburg, Würzburg, Germany.; 5 Department of Clinical Genetics, Erasmus MC, University Medical Centre Rotterdam, Rotterdam, Netherlands.

**Keywords:** Cell biology, Genetics, DNA repair, Genetic diseases, RNA processing

## Abstract

This study identifies and characterizes a novel type of splicing mutation in RAD50 deficiency, a rare genetic disorder.

**To the editor:** Human RAD50 deficiency is a very rare genomic instability syndrome associated with microcephaly and stunted growth (1). Here, we describe RAD50 deficiency in two siblings harboring a far-intronic *RAD50* 5 bp deletion (Δ5) in trans with a classic frameshift variant. The Δ5 mutation did not affect canonical splice sites but activated a poison 87 bp exon 30 nucleotides downstream. In the following, we provide evidence that this unusual pathogenic variant overcomes two blocking mechanisms to activate exon inclusion from a distance and a splice-switching strategy leads to effective correction.

Two siblings were diagnosed with clinical hallmarks of RAD50 deficiency at the Erasmus MC (Supplemental Methods and Supplemental Figure 1A; supplemental material available online with this article; https://doi.org/10.1172/JCI178528DS1). Genomic DNA sequencing of all RAD50 coding exons revealed heterozygosity for frameshift insertion c.2157_2158insT in both patients and their mother, and the RAD50 protein was almost absent in patient fibroblasts (Figure 1A and Supplemental Figure 1B). Subsequent *RAD50* cDNA sequencing revealed an unexpected 87-nucleotide insertion arising from intron 21 that preserved the reading frame while introducing a premature stop codon (Figure 1A). Surprisingly, no nucleotides immediately flanking this poison exon were mutated. Instead, we identified a TGAGT deletion (c.3390-1119_3390-1115del, NC_000005.10:g.132635996_132636000del) 30–34 nucleotides upstream of the cryptic acceptor site in both siblings and their father (Figure 1A and Supplemental Figure 1B). This suggested a noncanonical activation mechanism for the poison exon.

We designed a *RAD50* minigene construct to investigate how the Δ5 variant promotes exon inclusion (Supplemental Methods). Splicing pattern analysis by RT-PCR revealed that exon 21 was spliced to exon 22 for WT sequence, whereas the 5-nucleotide deletion resulted in poison exon inclusion (Figure 1B). This observation also held true when the *RAD50* sequence was placed into the context of a hybrid F9 minigene (Supplemental Figure 2) (2). These results established a causative role of the distant deletion for the splicing defect.

For mechanistic understanding, we performed site-directed mutagenesis of the TGAGT sequence (Figure 1C and Supplemental Figure 3). Any 3- or 4-base deletion activated the poison exon (Supplemental Figure 3). Single-base deletion of either A or G in positions 3 and 4 mimicked deletion of the entire element, while deletion of other bases induced only partial exon inclusion (Figure 1C). It made a difference whether GA or AG was retained (Del2 versus Del4), consistent with an AG-exclusion zone (AGEZ) (3). Among single-base substitutions, the strongest exon activation occurred when transverting positions 3 and 4 to pyrimidines while purine transitions showed attenuated effects (Figure 1C). Thus, although the AG dinucleotide seemed important in repressing the poison exon, additional elements contributed to the final block. This was further supported by the observation that the Del1,2 deletion induced exon activation up to approximately 50%, despite the presence of the AG dinucleotide (Figure 1C). As the most deleterious point mutations created pyrimidines, we next considered whether polypyrimidine tract (PPT) strengthening might contribute to exon activation, possibly through binding of splicing factors.

Two pyrimidine-rich sequences flank the TGAGT element. We observed significantly (*P* < 0.0001, unpaired *t* test, 2 degrees of freedom) more exon inclusion in minigene constructs bringing these imperfect pyrimidine stretches closer together, with less than 3 separating purines, although an AG partially suppressed this effect (Figure 1D). We evaluated the contribution of either imperfect PPT in separate mutagenesis experiments (Figure 1E). An enrichment of thymidines empowered the downstream PPT and activated poison exon inclusion despite the presence of the TGAGT element (3′-splice site–improved [3′ss-improved] construct, Figure 1E). Deletion of the upstream pyrimidine-rich sequence was neutral on the WT background (ΔTTTAT-WT) but reverted the effect of the original Δ5 deletion (ΔTTTAT-Δ5) (Figure 1E). Thus, distant exon activation required upstream pyrimidines to overcome a poor downstream PPT.

We then analyzed 11 factors known to affect 3′ss recognition in minigene cotransfection experiments. U2AF35 and U2AF65 strongly induced exon inclusion (Figure 1F). In vitro binding experiments with biotinylated RNA oligonucleotides and nuclear extracts indicated increased binding of U2AF65 to the Δ5 mutant and to a Δ3(GAG) core mutant RNA (Figure 1G). Thus, the most conceivable mechanism by which the Δ5 deletion promotes the aberrant 3′ss is by improving the affinity of the PPT to U2AF65 (Figure 1H).

To test functional correction strategies, we generated a large-T immortalized cell line from patient skin fibroblasts (F601-T). Like primary fibroblasts, F601-T cells expressed very low levels of RAD50 (Figure 1I). Complementation with WT *RAD50* (4) restored RAD50 and NBN protein levels and normalized radiation-induced phosphorylation of KAP1 and CHEK2 at ATM target sites (Supplemental Figure 4). We tested whether RAD50 levels could also be restored through targeted splicing correction with antisense morpholino oligonucleotides (AMOs). AMOs against either canonical splice site of the poison exon effectively suppressed exon inclusion (Figure 1I). Importantly, RAD50 protein levels and ATM kinase activity were restored, providing functional evidence of the AMO efficacy (Figure 1I) and suggesting a potential therapeutic strategy (5).

In conclusion, a far-intronic deletion in *RAD50* can cause disease through activation of an exon 30 nucleotides apart from the mutation site. Activating mutations generated a composite pyrimidine-rich sequence with increased U2AF binding. Exon inclusion required the shortening of an insulating stretch of 3 purines and was partly prevented by a residual AG dinucleotide. AMO-mediated splice switching fully restored normal RAD50 expression in patient-derived fibroblasts. Our findings help to characterize human RAD50 deficiency and uncover an unusual splicing mutational mechanism with potential relevance for other human genetic diseases.

## Supplementary Material

Supplemental data

Unedited blot and gel images

Supporting data values

## Figures and Tables

**Figure 1:**
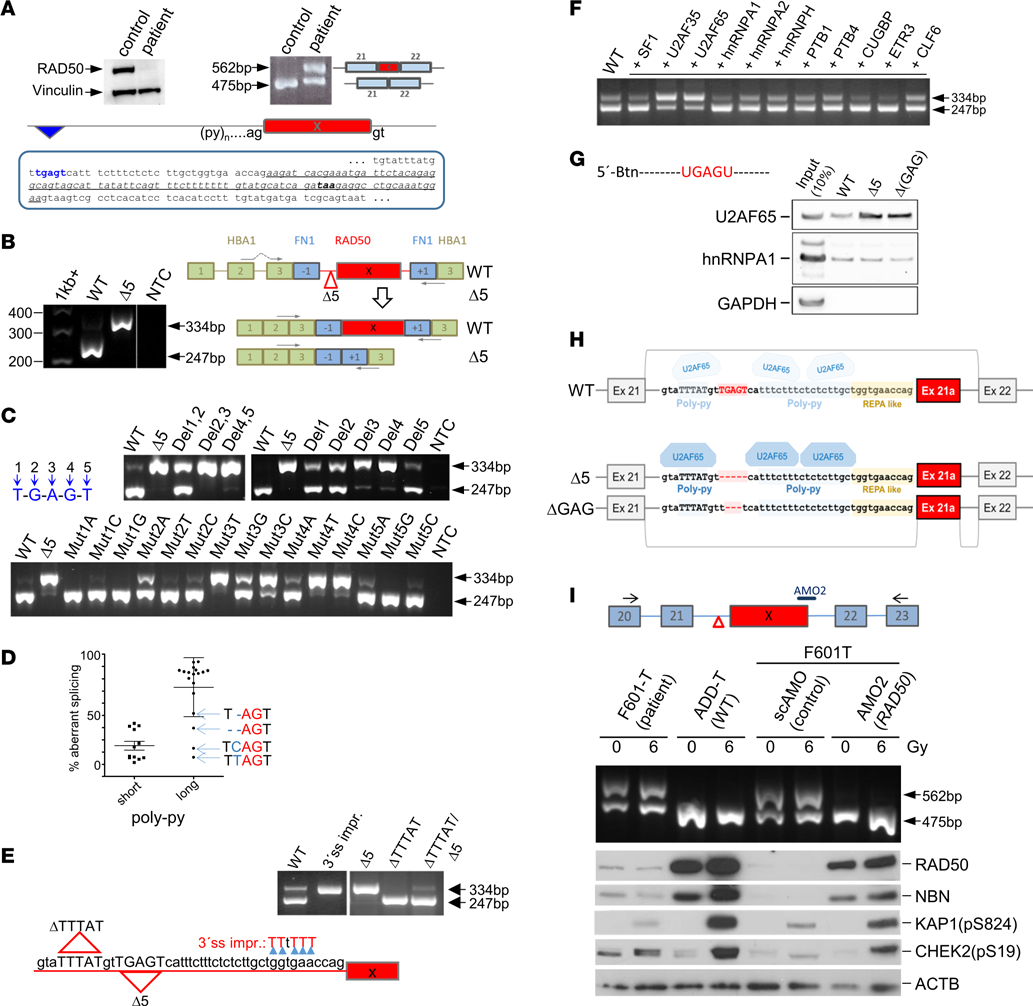
Pathogenic RAD50 splicing mechanism. (**A**) Top: Western blot of RAD50 protein (left) and RT-PCR of *RAD50* transcript (right) from control and patient fibroblasts. Bottom: Intron 21 with c.3390-1119_3390-1115del deletion (blue) and downstream poison exon (italics underlined). (**B**–**G**) Splicing analysis in HEK293 cells (see Supplemental Methods for additional information). (**B**) *RAD50* minigene constructs confirming exon inclusion for the Δ5 mutant. (**C**) Effects of deletions and substitutions within 5′-TGAGT-3′ on minigene splicing. (**D**) Exon inclusion with short versus long PPTs. Data are shown as the mean ± SD. (**E**) Role of pyrimidine-rich sequences for minigene splicing. (**F**) Overexpression of U2AF promotes aberrant splicing. (**G**) In vitro binding of U2AF to RNA oligonucleotides spanning the Δ5 region. (**H**) Model for a role of distal extension of the poly-pyrimidine region in promoting exon inclusion through U2AF accumulation, if devoid of an AG. (**I**) Correction of RAD50 splicing, protein level, and ionizing radiation–induced ATM signaling by AMO treatment.

## Version 1 12/12/2024

In-Press Preview

## Version 2 02/03/2025

Electronic publication
