# Supplementary material for: A deep intronic mutation causes RAD50 deficiency through an unusual mechanism of distant exon activation
Source: J Clin Invest. 2024 Dec 12;135(3):e178528. doi: 10.1172/JCI178528 (PMC11785915; doi:10.1172/JCI178528)
Supplement: Supplemental data [file jci-135-178528-s117.pdf]

A deep intronic mutation causes RAD50 deficiency through an unusual mechanism of distant exon activation

Patients and methods

Sex as a biological variable

Our study examined two sisters with a very rare disease. We did not statistically compare female and male patients because there is only one male patient with RAD50 deficiency in the current literature.

Clinical case reports

The index patient, a now 18-year-old female, was born after normal pregnancy and delivery at term with a low birth weight of 2090 grams (<-2 SD). Her exact birth length is unknown but at the age of one month, her length was 47 cm (-3.2 SD). At the age of one and a half years, she was seen by a pediatrician because of her short stature. Dysmorphic features were noted including a broad forehead with frontal bossing, micrognathia, small midface, short palpebral fissures, and translucent skin with prominent veins on the forehead. Since birth, she showed multiple café-au-lait spots with irregular borders and unpigmented flecks distributed over the skin of her whole body, which increased both in size and number with age. She presented with brachydactyly, bilateral clinodactyly of her fifth fingers, hypoplasia of the thumbs and thenars, and syndactyly of the second and third toes. Radiography demonstrated a left-sided brachymesophalanx of the fifth ray and synostosis of the *os trapezium* and *os scaphoideum*. The total level of immunoglobulins and subclass screening were normal. At the age of 10 years, medication with leuprorelin was started for precocious puberty and was continued till the age of 13 years and 7 months. At the last examinations, her head circumference was 51 cm at 14 years and 7 months (-2.3 SD), her height was 149.7 cm (-2.8 SD) and her weight 44.8 kg (+1.2 SD) at 15 years 8 months. Dermatologic

examination revealed pigment shifts in sun-damaged skin with multiple erythematous maculae as well as multiple lentigines in addition to the above-mentioned hyper- and hypopigmented spots.

Her younger sister was born after 38 4/7 weeks of gestation, likewise small for age with a birth weight of 1885 grams and unrecorded birth length. Polyhydramnios due to esophageal atresia with a TE fistula was noticed after delivery, for which she was operated two days after birth and later had several dilatations of the esophagus. Growth failure continued postnatally with a height following a growth curve of -3 SD. She was not microcephalic and had a small head circumference at -1.7 SD. Her facial features were similar to her older sister, and she likewise revealed café-au-lait spots of the skin, hypoplastic thumbs and thenars, brachydactyly and bilateral clinodactyly of both digits V of the hands. On radiography of her left hand, she also showed a short middle phalanx of the fifth ray. At the last examinations at the age of 13 years and 9 months her height was 140.8 cm (-3,36 SD) and her weight 32.3 kg (-2,9 SD).

At their present age of 18 and 16 years, respectively, both siblings are devoid of recurrent infections, have normal IgG/IgA levels, and no tumors. They both have a high-pitched voice. The main clinical features of the two siblings in comparison to previously reported patients with RAD50 deficiency (Refs. 1,6,7,8) are summarized in **Supplementary Figure 1A**.

Sequencing analyses

Genomic DNA was isolated from peripheral white blood cells of the two siblings and their parents as well as from the established fibroblast line by using a QIAmp blood kit (Qiagen). Coding exons were analyzed from the patient´s sample using Haloplex sequencing (Ref. 9). Mutation confirmation was performed by direct sequencing of the PCR products using BigDye Terminator chemistry (Applied Biosystems) and capillary electrophoresis with the SeqStudio Genetic Analyzer. Targeted RNA sequencing was performed after full-length amplification of the *RAD50* transcript. The *RAD50* transcript was fragmented by sonication using a bioruptor instrument

(Diagenode) and sequenced on a Miseq platform (Illumina Inc.) with the 100 bp paired-end protocol. Raw sequence data were aligned to human reference genome hg19 with bwa (Ref. 10) to generate the bam files which were visualized in IGV (https://software.broadinstitute.org/software/igv/). For cDNA sequencing, total RNA was extracted using RNAzol reagent and reverse transcribed using a First Strand cDNA Synthesis Kit (New England BioLabs). RT-PCR products from *RAD50* transcripts were obtained with the primers 5´-GAAAGGTGGCTACAAGATAAC-3´ (exon 20) and 5´-CATTTTCATCGGCATCAGACCG-3´ (exon 23) which yielded products of 475 bp for the wild-type splicing and 562 bp for the aberrant splicing including the cryptic exon. The RT-PCR products were sequenced using BigDye Terminator chemistry (Applied Biosystems) and capillary electrophoresis with the SeqStudio Genetic Analyzer.

Cell culture and treatment

A fibroblast cell line was established from a skin biopsy of the elder patient and was immortalized using nucleofection with SV40 largeT plasmid as previously described (Ref. 1). These cells, referred to as F601-T, were cultured in IMEM with 15% heat-inactivated fetal calf serum, 100 U/ml penicillin, 0.1 mg/ml streptomycin and 2 mM L-glutamine. A normal control fibroblast line, ADD-T, from a healthy 23-year-old female and the fibroblast line from the original female patient with RAD50 deficiency, F239-T, had been previously established in the same way (Refs. 1,4). Human embryonic kidney cells HEK293T were kindly provided by Axel Schambach (Experimental Hematology, MHH). ADD-T, F239-T and HEK239T cells were cultured in DMEM (high glucose) with 10-15 % heat-inactivated fetal calf serum, 100 U/ml penicillin and 0.1 mg/ml streptomycin. All cells were maintained at 37 °C in a humidified atmosphere supplemented with 5 % CO_2. All cells were regularly PCR-tested to be free from mycoplasma.

For irradiation experiments, ionizing radiation (6Gy) was applied to the cells by a Mevatron MD-2 accelerator in the Clinics of Radiation Therapy at Hannover Medical School. Complementation

of the F601-T cells with RAD50 was performed with a lentiviral vector expressing codon-optimized *RAD50*, in comparison with an empty vector control as we have described previously (Ref. 4). For antisense treatment, two 25-mer antisense morpholino oligonucleotides (AMOs) were designed to target the 5′ and the 3´ -sites of the aberrant exon embedded in intron 21 of the *RAD50* pre-mRNA. The AMO1 targeting the acceptor splice site within intron 21A was 5´-ATCTTCTGGTTCACCAGCAAGAGAG-3´, and the AMO2 targeting the donor splice site within intron 21B was 5´-AGGATGTGAGGCGACTTACTTCCAT-3´ (bases complementary to donor or acceptor dinucleotides underlined). As AMO2 was slightly more efficient than AMO1, only AMO2 was used for subsequent irradiation experiments (Figure 1I). A standard scrambled AMO (scAMO) (5´- CCTCTTACCTCAGTTACAATTTATA-3´) served as non-specific control. AMOs were synthesized by Gene-Tools (Philomath, OR). For scrape delivery of AMOs, 3-5 x 10^5 immortalized patient fibroblast cells per well were seeded into a six-well plate. After 24 h, AMOs were added directly to the medium at a final concentration of 10 µM, and cells were carefully scraped with a rubber device and plated into a fresh well to allow uptake. Cells were collected after 24, 48, and 72 h for RNA analysis (from 1 well each), and after 70-72 h for RAD50 protein detection (from 2 wells combined). Very similar results were obtained when the AMOs were delivered by nucleofection (not shown).

Design and cloning of minigene constructs

To investigate the molecular mechanisms of *RAD50* intron 21 splicing in an established splicing system, a region of the intron 21 sequence spanning from 396/391 bp upstream to 277 bp downstream of the silent exon was amplified from the chromosomal DNA isolated from the patients immortalized fibroblasts and cloned into the plasmid pTB NdeI, a previously described minigene plasmid derived from a pBlueScript backbone, which harbors hybrid *HBA1-FN1* sequences upstream and downstream of the insertion site (Refs. 2,11). Sequence analysis of the *RAD50* portion was performed to verify pTB-NdeI-*RAD50*-WT and pTB-NdeI-*RAD50*-Δ5.

Mutant sequences were generated either by overlapping PCR using the mutagenesis primers listed below (Mut3C, Mut3G, Mut3T) or were purchased from Genescript (all others). Three of the mutations represented naturally occurring single-nucleotide variant listed in the NCBI SNP database: rs1010201800 (Mut3C), rs1328381005 (Mut4A) and rs1206337881 (Mut1G). All *RAD50*-derived sequences were confirmed by Sanger sequencing. Plasmids were prepared using the DNA Plasmid Purification NucleoBond® Xtra Kit (Macherey-Nagel). Full sequences of selected plasmids are available as .ape files upon request.

Primers used in this study

The table lists the oligonucleotide sequences of primers used for mutagenesis, sequencing or RT-PCR as specified in the "purpose" column

**Name**	**Sequence 5´-3´**	**Purpose**
R50 minigene 601NdeI F	AAAAAACATATGCTGCCTTGTCTGGAGCTTG	cloning, mutagenesis and sequencing
R50 minigene 601NdeI R	AAAAAACATATGAAGCACTAAATTCCACTTAGTCCAG	cloning, mutagenesis and sequencing
R50 minigene Mut3_F	GATATTTAAAATGTATTTATGTTGBGTCATTTCTTTCTCTCTTGCTGG	mutagenesis
R50 minigene Mut3_R	GAGAAAGAAATGACVCAACATAAATACATTTTAAATATCAGATGAAGG	mutagenesis
MinigeneSeq F1	TTCCTCAGCCTGAATGTGC	sequencing
R50 minigene SeqF	TGGTGAACCAGAAGATCACG	sequencing
R50 minigene SeqR	TCTTTGGAAGCGGGTTTATG	sequencing
R50 minigene SeqR2	GAAGGACTGTGAAGTAGTAGAAGAC	sequencing
R50MiniSeqMut	GGAGACTTGTTGCATTCTCATC	sequencing
RAD50ex20F	GAAAGGTGGCTACAAGATAAC	cDNA analysis endogenous
RAD9R(RAD9-2)	CATTTTCATCGGCATCAGACCG	cDNA analysis endogenous
α2,3 dir	CAACTTCAAGCTCCTAAGCCACTGC	cDNA analysis minigene
α2,3 dir-EcoRI	AAAGAATTCCAACTTCAAGCTCCTAAGCCACTGC	cDNA analysis minigene
Bra2 rev	TAGGATCCGGTCACCAGGAAGTTGGTTAAATCA	cDNA analysis minigene

Cell transfections and minigene splicing assay

HEK239T cells were used for the splicing minigene assays and transfected either by PEI (Polyscience, 0.1 g/l 150 mM NaCl, pH 5.5) with 1.3 μg minigene plasmids and 0.13 ug GFP control plasmid (Figure 1B-E), by Effectine (Qiagen) or by Lipofectamine 2000 (Invitrogen) using 0.5 µg minigene plasmid and 0.5 μg plasmid for exogenous expression of splice factors (Figure 1F). After 24 h, total RNA was extracted and RT was performed using 2 µg of RNA, and the cDNA (Protoscript II, New England BioLabs) was used in PCR with *Taq* DNA Polymerase (Qiagen). Primers used to amplify minigenes were "α2,3 dir" (or "α2,3 dir-EcoRI") and "Bra2 rev", as listed above. PCR products were separated on a 2% agarose gel and the intensity of the bands was measured with ImageJ (Ref. 12). Selected samples were additionally amplified with Bra2 rev and a FAM-labelled α2,3 dir primer, mixed with ROX-labelled size standard (GeneScan 500 ROX; Applied Biosystems), denatured and run on a SeqStudio capillary electrophoresis instrument (Applied Biosystems). The area under the peaks as calculated by the Fragment Analysis software was consistent with Image J calculations from agarose gels.

RNA-oligo pulldown

Three synthetic biotinylated RNA oligonucleotides (Sigma-Aldrich) were used for pulldown assays: *RAD50* WT (5'-[Btn] AUUUAUGUUGAGUCAUUUCU-3'), *RAD50* Δ5 (5'-[Btn] AUUUAUGUCAUUUCUUUCUC-3') and *RAD50* ΔGAG (5'-[Btn] AUUUAUGUUUCAUUUCUUUC-3'). To capture RNA oligo-protein complex, M-280 streptavidin Dynabeads (Invitrogen) together with DynaMag-Spin Magnet (Invitrogen) were used following the manufacturer´s instructions. Solutions and buffers were used as previously published by Giulia Romano and colleagues (Ref. 13). Briefly, 1 mg streptavidin-coated slurry beads were incubated with biotinylated snRNAs for 15 minutes RT in B&W buffer (2X: 10 mM Tris HCl pH 7.5, 1 mM EDTA, 2 M NaCl). After washes, 500 ug HeLa cells nuclear extracts were incubated

with biotinylated snRNAs in HEGN buffer (2X: 40 mM Hepes pH 7.7, 300 mM NaCl, 1 mM EDTA, 20% Glycerol, 0.2% Triton-X100, 2 mM DTT), with fresh protease-inhibitor tablet (Roche) and 0.2 U/µL RNasin RNase inhibitor (Promega) and heparin (final concentration, 0.5 µg/µl) for 1 h at 4°C on a rotator. Bound proteins on the beads were eluted by boiling in 40 µL of 1× loading dye sample buffer, resolved by SDS-PAGE and detected by western blotting.

Immunoblotting

Western blots of total protein extracts were performed as previously published (Ref. 4). Briefly, cells were lysed in cell lysis buffer (50 mM Tris pH 7,4, 150 mM NaCl, 2 mM EGTA, 2 mM EDTA, 25 mM NaF, 0.1 mM Na_3VO_4, 0.1 mM PMSF, 2 mg/ml Leupeptin, 2 mg/ml Aprotinin, 0.2 % Triton X-100, 0.3 % Nonidet P-40) for 30 min on ice and centrifuged at 16,100 rcf for 15 min. After the addition of the loading buffer, protein extracts were separated through SDS-PAGE and blotted onto nitrocellulose membranes (GE Healthcare, Amersham Hybond ECL). The following primary antibodies were used: monoclonal mouse anti-RAD50 (Abcam, ab89); polyclonal rabbit anti-NBN (Gentex, GTX103229), rabbit polyclonal anti-CHEK2-pS19 (CellSignaling #2666), rabbit polyclonal anti-KAP1-pS824 (Bethyl A300-767), mouse monoclonal anti-vinculin (Santa Cruz sc-25336), anti-β-actin (Sigma A5541), U2AF65 (1:1000, ICGEB home-made), hnRNPA1 (1:1000, ICGEB home-made) and GAPDH (1:6000, Abcam). Anti-mouse and anti-rabbit horseradish peroxidase-labeled secondary antibodies were purchased from GE Healthcare or Agilent (#P044701-5 and # P044801-2, respectively and Dako P0447012). Enhanced chemiluminescence (Dura ECL, Thermo Scientific/Pierce, or Amersham, Les Ulis, France) was used for the visualization of immunoreactive bands either by film or using an Uvitec imaging system (Cambridge, UK).

Statistical analysis and bioinformatics

The *RAD50* sequence was based on the NCBI Gene sequence NT_034772.5 in which the five-base-pair deletion is located at nucleotides 34386701-34386705 on chromosome 5. We analyzed splice site scores for the acceptor and donor sites of the cryptic exon 21a and the canonical exon 22 using MaxEntScan in the default maximum entropy mode (http://hollywood.mit.edu/burgelab/maxent/Xmaxentscan_scoreseq_acc.html; Ref. 14).

Statistical analysis between three or more groups was performed by ANOVA or mixed-effects model (REML) analysis in GraphPad Prism v9.5.1. Multiple comparisons were performed between groups and p-values were adjusted using Tukey's multiple comparisons test after ANOVA or Bonferroni correction after REML. For RNA oligo-pulldown experiment, One-way ANOVA followed by Sidak multiple comparison test was performed using GraphPad Prism.

Study approval

Written informed consent for the mutational study and for the publication of scientific results was received from the index family at the University Medical Center Rotterdam. The project was part of the work on genomic instability disorders approved by the Institutional Review Boards of the Faculty of Medicine at the University of Würzburg and at Hannover Medical School.

Data availability

Whole sequence information for the plasmids used are available as .ape files upon reasonable request. Supporting data values for all graphs and statistical analyses can be found in an accompanied XLS file.

Acknowledgments

We are grateful to the index family for participating in this study. We thank Giulia Pianigiani, Fabienne Hülse and Martin A. Rooimans for technical assistance, Marjolein Willemsen for helpful hints, and the Claudia von Schilling Foundation for their financial support. We gratefully remember the help of Johan de Winter at the UMC Amsterdam in identifying these patients and setting up this collaborative effort between our centers. We thank Josephine Dorsman and Hans Gille at the UMC Amsterdam for their further support. We thank Cristiana Stuani, Raffaella Klima and Giulia Romano from ICGEB (Trieste, Italy) to have generously provided us with antibodies for U2AF65 and hnRNPA1 with DynaMag-Spin Magnet (Invitrogen), respectively.

Author contributions

KB, SD, TF and MP performed the splicing minigene experiments. KB and TF performed the AMO experiments. SD performed the experiments with splicing factors and *in vitro* binding studies. NA performed the diagnostic sequencing and the cDNA analyses which initially identified the mutation. DR performed bioinformatics analyses. DS established the fibroblast line and initiated the study. IL performed the clinical data analysis. FP and TD designed the study. KB, SD, FP and TD analyzed the splicing results. KB and TD drafted the manuscript. All authors read and approved the final version of the manuscript.

References

1. Waltes R, et al. Human RAD50 deficiency in a Nijmegen breakage syndrome-like disorder. Am J Hum Genet. 2009;84(5):605-616.

2. Tajnik M, et al. Molecular Basis and Therapeutic Strategies to Rescue Factor IX Variants That Affect Splicing and Protein Function. PLoS Genet. 2016;12(5):e1006082.

3. Wimmer K, et al. AG-exclusion zone revisited: Lessons to learn from 91 intronic NF1 3'-splice site mutations outside the canonical AG-dinucleotides. Hum Mutat. 2020;41(6):1145-1156.

4. Völkening L, et al. RAD50 regulates mitotic progression independent of DNA repair functions. FASEB J. 2020;34(2):2812-2820.

5. Kim J, et al. A framework for individualized splice-switching oligonucleotide therapy. Nature. 2023;619(7971):828-836.

6. Ragamin A, et al. Human RAD50 deficiency: Confirmation of a distinctive phenotype. *Am J Med Genet A.* 2020;182(6):1378-1386.

7. Chansel-Da Cruz M, et al. A Disease-Causing Single Amino Acid Deletion in the Coiled-Coil Domain of RAD50 Impairs MRE11 Complex Functions in Yeast and Humans. *Cell Rep.* 2020;33(13):108559.

8. Takagi M, et al. Bone Marrow Failure and Immunodeficiency Associated with Human RAD50 Variants. *J Clin Immunol*. 2023 Nov; 43(8):2136-2145.

9. Naipal KA, et al. Functional ex vivo assay to select homologous recombination-deficient breast tumors for PARP inhibitor treatment. *Clin Cancer Res.* 2014;20(18):4816-4826.

10. Li H, Durbin R. Fast and accurate short read alignment with Burrows-Wheeler transform. *Bioinformatics*. 2009;25(14):1754-1760.

11. Baralle M, et al. Identification of a mutation that perturbs NF1 gene splicing using genomic DNA samples and a minigene assay. *J Med Genet.* 2003;40:220-222.

12. Schneider C, Rasband W, Eliceiri K. NIH Image to ImageJ: 25 years of image analysis. *Nat Methods* 2012;9:671–675.

13. Romano G, Klima R, Feiguin F. Immunoprecipitation for Protein-Protein Interactions and for RNA Enrichment in Drosophila melanogaster. *Bio Protoc*. 2021;11(23):e4250.

14. Yeo G, Burge CB. Maximum entropy modeling of short sequence motifs with applications to RNA splicing signals. *J Comput Biol.* 2004;11(2-3):377-394.

Supplemental Figure1: Clinical and mutation description of the index patients

A. Clinical phenotypes of the index siblings and comparison to previously reported patients with RAD50 deficiency

**Phenotype**	**Patient 1 and 2** (This study)	**RAD50 deficiency/ NBSLD** (Waltes et al. 2009)	**RAD50 deficiency/ NBSLD** (Ragamin et al. 2020)	**RAD50 deficiency** (Chansel-Da Cruz et al., 2020)	**RAD50 deficiency** (Takagi et al., 2023)
Gene variant	c.2157_2158insT/ c.3390-1119_3390-1115del	c.3277C>T/ c.3939A>T	Homozygous c.2524G>A	c.2165dup/ c.3109_3111del	c.248G>A/ c.1453G>T
Protein change	p.Glu723Glyfs*5/ p.Glu1130insTer*23	p.Arg1093Ter/ p.Ter1313TyrextTer*66	p.Met800Phefs*7 (minor p.Val842Ile)	p.Glu723Glyfs*5/ p.Glu1035del	p.Arg83His/ p.Glu485X (or p.Glu485_Lys545del)
Age at first description	18 ys / 16 ys	23 y	15 y	17 y	9 y
Dysmorphic facial features	+	+	+	+	+
Short stature	-2.7 / -3.4 SD	< -3 SD	< - 3.5 SD	< -3 SD	< -2 SD
Head circumference	-2.3 / -1.7 SD	< -3 SD	< - 3.5 SD	< -3 SD	< -3 SD
Immunodeficiency	-	-	-	Very low B-cell count, impaired T lymphopoiesis	Hypogammaglobulinemia, low T-cell counts, absence of B-cells
Bone marrow failure	-	-	-	+	+
Skin pigmentation anomalies	+	+	+	+	-
Cancer	-	-	-	-	-
Ataxia	- / -	-	-	-	-
Intellectual development	Above average	Delayed	Initial delay in speech. At age 15, TIQ estimated 85.	?	?
Sexual development	Precocious puberty	Normal puberty	Normal puberty	?	Death by age 9
Further manifestations	Esophageal atresia, digital abnormalities	Hyperopia, high-pitched voice	Hearing loss	Anemia, dental loss, cataract	Cardiomyopathy, epilepsy

B. Identification and family segregation of pathogenic *RAD50* variants

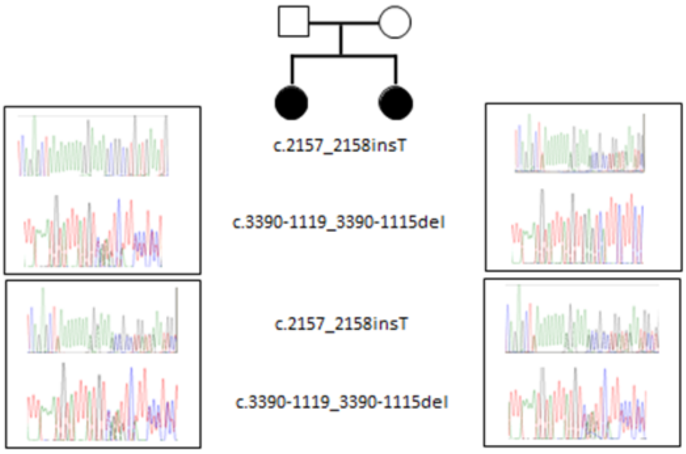

Legend to Supplemental Figure 1: Clinical and mutation description of the index patients

A. Clinical phenotypes of the index siblings and comparison to previously reported patients with RAD50 deficiency (Refs. 1,6,7,8). Clinical phenotypes for the two siblings identified in this study are listed in column 2 (for more details see also description in the Patients and Methods section), in comparison to the four other patients with RAD50 deficiency published previously (columns 3-6). NBSLD, Nijmegen Breakage-like disorder; SD, standard deviation; n.d., not determined; TIQ, total intelligence quotient.

B. Identification and family segregation of frameshift variant c.2157_2158insT (upper sequence per individual, exon 13) and far-intronic deletion c.3390-1119_3390-1115del (lower sequence, intron 21) in the *RAD50* gene. Upper left: Father, heterozygous for the intronic deletion c.3390-1119_3390-1115del. Upper right: mother heterozygous for the frameshift insertion c.2157-2158insT. Bottom left and right: the two siblings compound heterozygous for both mutations.

Supplemental Figure 2: *RAD50* minigene splicing analysis

A. Transposition of *RAD50* 3´splice region to unrelated exon

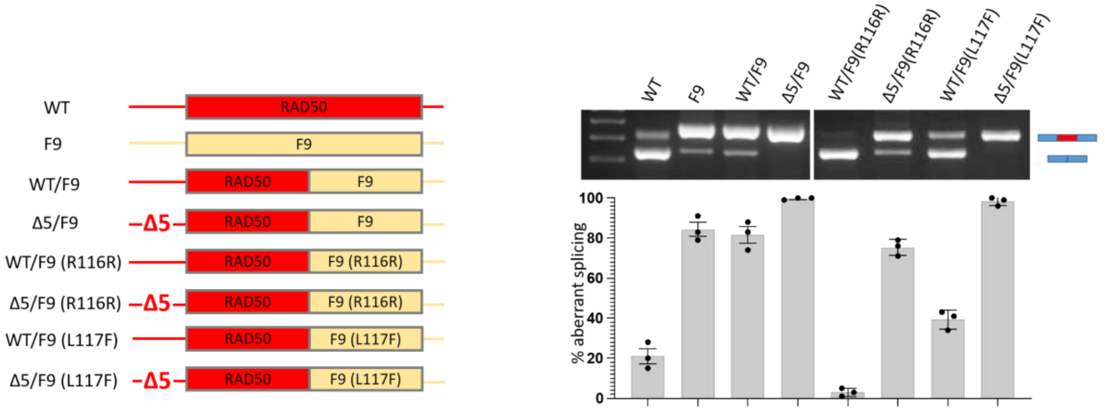

B. Mutants generated within the Δ5 region of *RAD50* minigene

WT	5´-tatttatgt **TGAGT** catttctttctctcttgctggtgaacc*ag*
Δ5	5´-tatttatgt ----- catttctttctctcttgctggtgaacc*ag*
Mut1A	5´-tatttatgt **AGAGT** catttctttctctcttgctggtgaacc*ag*
Mut1C	5´-tatttatgt **CGAGT** catttctttctctcttgctggtgaacc*ag*
Mut1G	5´-tatttatgt **GGAGT** catttctttctctcttgctggtgaacc*ag*
Mut2A	5´-tatttatgt **TAAGT** catttctttctctcttgctggtgaacc*ag*
Mut2C	5´-tatttatgt **TCAGT** catttctttctctcttgctggtgaacc*ag*
Mut2T	5´-tatttatgt **TTAGT** catttctttctctcttgctggtgaacc*ag*
Mut3C	5´-tatttatgt **TGCGT** catttctttctctcttgctggtgaacc*ag*
Mut3G	5´-tatttatgt **TGGGT** catttctttctctcttgctggtgaacc*ag*
Mut3T	5´-tatttatgt **TGTGT** catttctttctctcttgctggtgaacc*ag*
Mut4A	5´-tatttatgt **TGAAT** catttctttctctcttgctggtgaacc*ag*
Mut4C	5´-tatttatgt **TGACT** catttctttctctcttgctggtgaacc*ag*
Mut4G	5´-tatttatgt **TGAGT** catttctttctctcttgctggtgaacc*ag*
Mut5A	5´-tatttatgt **TGAGA** catttctttctctcttgctggtgaacc*ag*
Mut5C	5´-tatttatgt **TGAGC** catttctttctctcttgctggtgaacc*ag*
Mut5G	5´-tatttatgt **TGAGG** catttctttctctcttgctggtgaacc*ag*
Del1,2,3,4	5´-tatttatgt **----T** catttctttctctcttgctggtgaacc*ag*
Del1,2,3	5´-tatttatgt **---GT** catttctttctctcttgctggtgaacc*ag*
Del2,3,4	5´-tatttatgt **T---T** catttctttctctcttgctggtgaacc*ag*
Del3,4,5	5´-tatttatgt **TG---** catttctttctctcttgctggtgaacc*ag*
Del1	5´-tatttatgt **–GAGT** catttctttctctcttgctggtgaacc*ag*
Del2	5´-tatttatgt **T-AGT** catttctttctctcttgctggtgaacc*ag*
Del3	5´-tatttatgt **TG-GT** catttctttctctcttgctggtgaacc*ag*
Del4	5´-tatttatgt **TGA-T** catttctttctctcttgctggtgaacc*ag*
Del5	5´-tatttatgt **TGAG-** catttctttctctcttgctggtgaacc*ag*
Del1,2	5´-tatttatgt **--AGT** catttctttctctcttgctggtgaacc*ag*
Del2,3	5´-tatttatgt **T--GT** catttctttctctcttgctggtgaacc*ag*
Del4,5	5´-tatttatgt **TGA--** catttctttctctcttgctggtgaacc*ag*

C. Splicing of *RAD50* minigenes with distinct deletions in the Δ5 sequence

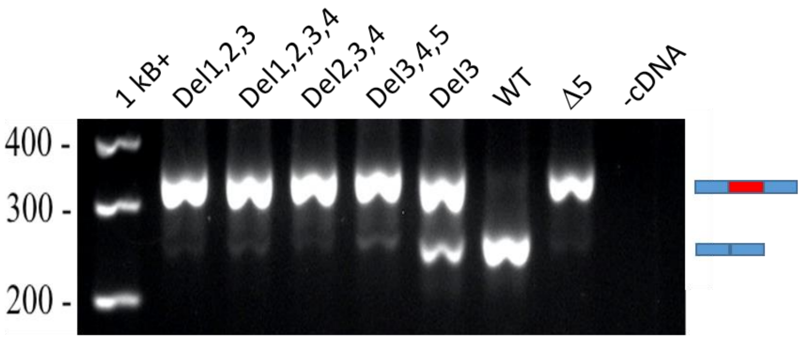

D. Relative proportion of exon inclusion in *RAD50* minigenes with deletion or substitution mutants within the Δ5 sequence (Fig. 1C)

Single-base deletions

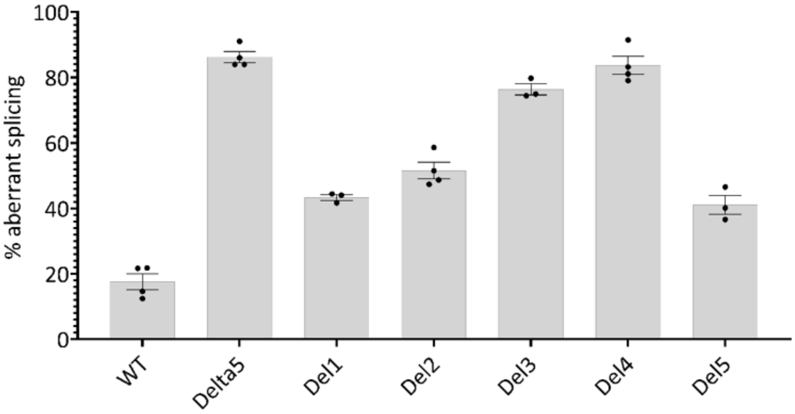

Two-base deletions

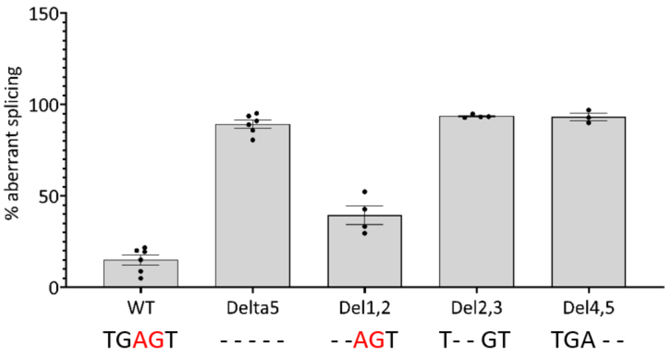

Single-nucleotide substitutions

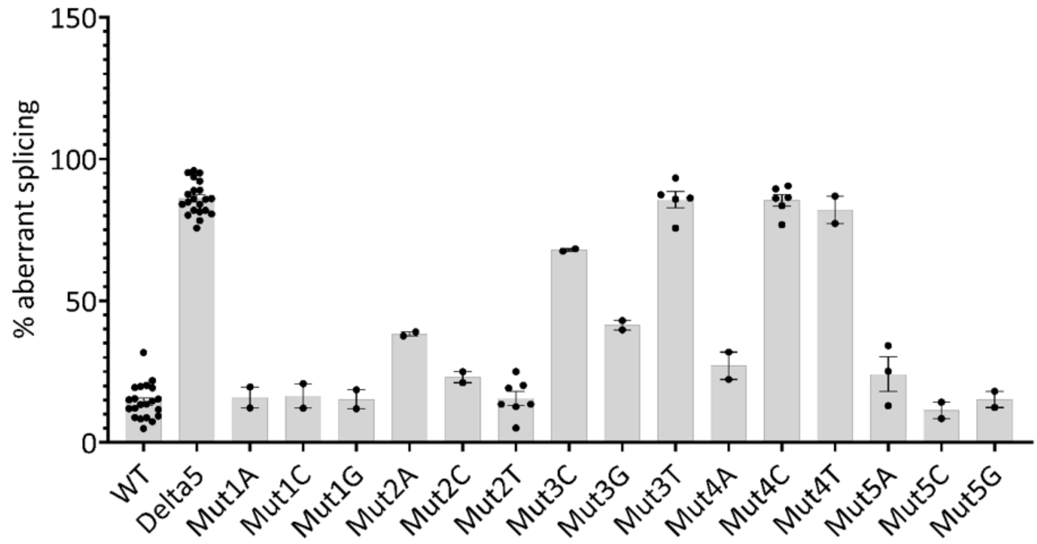

E. Relative proportion of exon inclusion for single-base deletions retaining AG versus GA in fragment length analyses

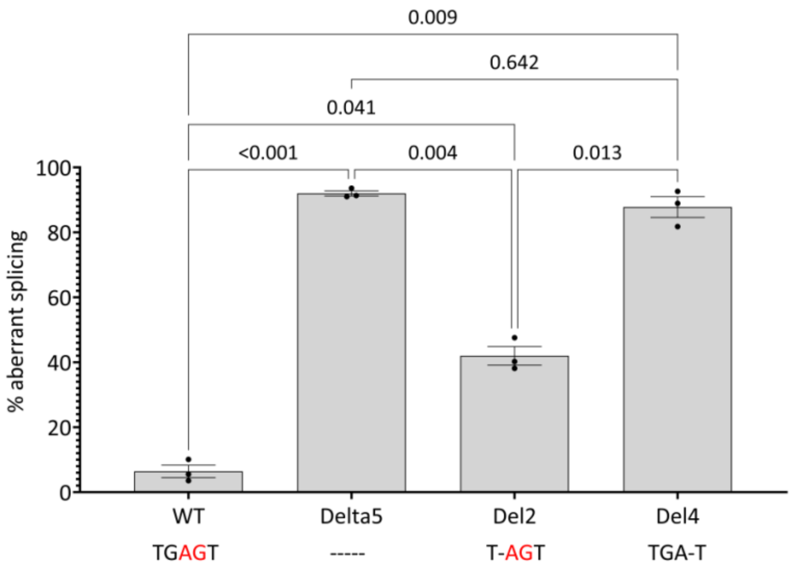

Legend to Supplemental Figure 2: *RAD50* Minigene splicing analysis

A. Transposition of *RAD50* 3´splice region to unrelated exon

Hybrid-FIX and Δ5-FIX minigene constructs were generated consisting of a *RAD50* sequence from –396 to +22 relative to the silent exon), fused to the exon 5 of the *F9* gene for Factor IX (starting from position +21). *RAD50*, *F9* and hybrid minigene constructs were transfected into HEK239T cells and splicing patterns were analyzed by agarose gel electrophoresis; n=3 for all conditions. Note that *RAD50* WT-*FIX* yielded a similar leaky splicing pattern as the construct with *F9* exon 5 only, whereas full exon inclusion was observed for the *RAD50* Δ5-*FIX* minigene. Splice-repressive point mutations, R116R and L117F, were introduced into *F9* exon 5 (see Methods section). These mutants were weakly or strongly suppressive, respectively, in the hybrid minigenes. This suppression can be overcome by the deletion of the 5 bp motif (*RAD50* Δ5-*FIX)*.

B. Mutants generated within the Δ5 region of *RAD50* minigene.

Sequence information is shown for each analyzed point and deletion mutant in the region around the Δ5 motif (bold). Mutated or deleted bases are marked in red. The AG at the end of intron 21 is indicated in italics. Three of the mutants represented naturally occurring single-nucleotide variant listed in the NCBI SNP database: rs1010201800 (Mut3C), rs1328381005 (Mut4A) and rs1206337881 (Mut1G).

C. Splicing of RAD50 minigenes with distinct deletions in the Δ5 sequence.

Minigene constructs including the four-base deletion, any three-base deletion or the deletion of the central A in the Δ5 region were transfected into HEK293T cells and total RNA was isolated 24 h later. After RT-PCR, splicing products were analyzed using agarose gel

electrophoresis. In the case of the RAD50 wild-type (WT) minigene, the poison exon was largely excluded, resulting in a 247 bp PCR product. In contrast, after transfection of the RAD50-Δ5 minigene (Δ5) a 334 bp PCR product indicated mainly exon inclusion (schematically illustrated at the right, with poison exon in red). All three- or four-base deletions similarly resulted in an efficient inclusion of the poison exon, which was also the main outcome of the single-base deletion of the central A within the Δ5 region (Del 3). 1 kB+, size marker; -cDNA, no template control.

D. Relative proportion of exon inclusion in *RAD50* minigenes with deletion or substitution mutants within the Δ5 sequence. Image J quantification was performed for all individual mutants shown in Figure 1C and a combined analysis of all biological replicates was conducted. The percentage of exon inclusion is shown for each mutant, with numbering according to its position in the 5´-TGAGT-3´ region. Error bars represent SEM. *Single-base deletions*: n=4 for WT, Δ5, Del2 and Del4; n=3 for Del1, Del3 and Del5. In comparison to WT and Δ5, the single deletion mutants Del3 and Del4 reached levels of exon inclusion that were indistinguishable from Δ5 (p=0.22 for Del3 and p>0.99 for Del4; Mixed-effects model (REML) with Bonferroni correction for multiple-comparison (n=11). *Two-base deletions*: n=6 for WT and Δ5, n=4 for Del1,2 and Del2,3, and n=3 for Del4,5. In comparison to WT and Δ5 mutation, the two-nucleotide deletion mutants Del2,3 and Del4,5 reached levels of exon inclusion that were indistinguishable from Δ5 (p=0.34 for Del2,3 and p=0.70 for Del4,5) while the Del1,2 mutant that retains the –AG- allowed only for some 50% splicing and remained significantly different from Δ5 (p=0.008 after correction for multiple comparisons (n=10)). *Single-nucleotide substitutions*: n=21 for WT and Δ5, n=7 for Mut2T, n=6 for Mut4C, n=5 for Mut3T, n=3 for Mut5A and n=2 for all others. After correction for multiple testing, Mut3T and Mut4C

remained significantly different from wildtype (p<0.001; Mixed-effects model (REML) with Bonferroni correction for multiple-comparison (n=29).

E. Relative proportion of exon inclusion for single-base deletions retaining AG versus GA. Fluorescence-based fragment length analysis after capillary electrophoresis was performed for a more quantitative comparison of the Del2 and Del4 mutants. Del4 (TGA-T, lacking an AG) supported exon inclusion comparable to Δ5, while Del2 (T-AGT, retaining the AG) allowed for less than 50% splicing and significantly differed to both Del4 and Δ5 (adjusted p=0.004 and p=0.013, respectively; n=3; ANOVA and Tukey´s Multiple Comparison Test for 6 comparisons). Note that Del2 is one of the four AG-retaining mutants highlighted in Figure 1D that showed reduced exon inclusion despite an extension of the poly-pyrimidine stretch: Del1,2 (--AGT), Del2: T-AGT, Mut2T (TTAGT) and Mut2C (TCAGT), though they still allowed for exon inclusion above the wild-type level. Overall, a distally extended poly-pyrimidine stretch with less than 3 consecutive purines promoted exon inclusion with high significance (p<0.0001; unpaired two-tailed t-test)(Fig. 1D).

Supplemental Figure 3: Role of PPT length for poison exon usage

A. Quantitative analysis of exon inclusion with mutated proximal or distal pyrimidine-rich regions

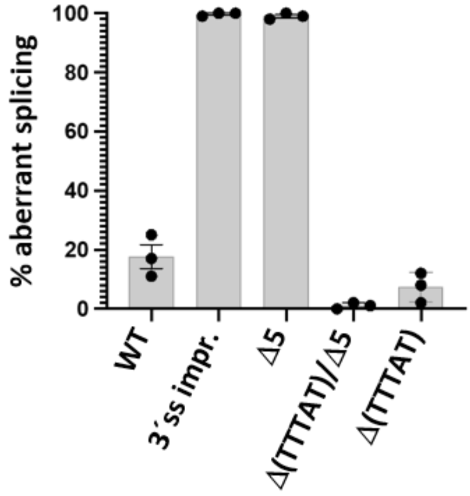

B. Quantitative analysis of exon inclusion after exogenous expression of respective splice factors

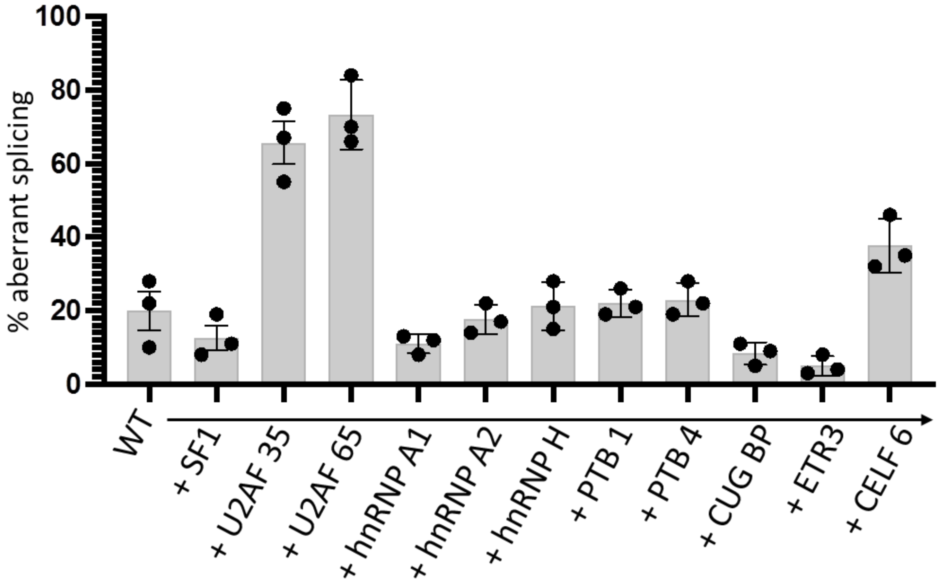

C. Quantification of in vitro U2AF65 pulldown with RNA-oligonucleotides spanning the ∆5 region

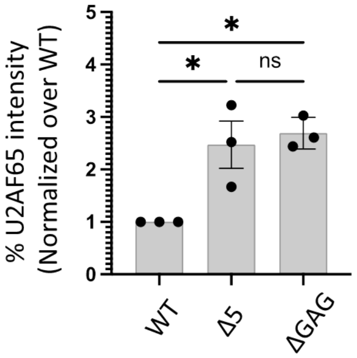

Legends to Supplemental Figure 3: Role of PPT length for poison exon usage

A. Quantification of splicing of RAD50 minigenes with mutated proximal and distal polypyrimidine-rich region, as shown in Figure 1E. An improved 3´splice site was generated by the transversion of five purines to pyrimidines in the proximal region of the otherwise WT RAD50 minigene. To assess the distal sequence, a TTTAT sequence just upstream of the ∆5 region was deleted in both the RAD50 wild-type and the ∆5 minigene sequence (Figure 1E). Improving the 3´splice site by an extended proximal pyrimidine tract after removal of blocking purines promoted efficient exon inclusion in the otherwise WT RAD50 minigene. Deletion of the TTTAT stretch in the distal pyrimidine-rich region did not activate the exon and suppressed the effect of the ∆5 mutant.

B. Quantitative analysis of exon inclusion after exogenous expression of respective splice factors. HEK293T cells were transfected with expression constructs for either of eleven known splicing factors (see Methods and Figure 1F). Exogenous expression of either U2AF35 or U2AF65 strongly promoted exon inclusion from the *RAD50* WT minigene.

C. Quantification of in vitro U2AF65 pulldown with RNA-oligonucleotides spanning the Δ5 region. Biotinylated RNA 20mer-oligonucleotides representing either the *RAD50* wild-type sequence, the Δ5 mutant or a Δ3-core mutant were used to assess *in vitro* binding (Figure 1G). The pulldown/binding assay was performed as described in the Methods section. A western blot was performed after the pulldown to investigate proteins bound to these RNA 20mers, and the quantification of U2AF65 intensity, normalized to wildtype, is shown. Compared to WT construct, U2AF65 binding to both Δ5 mutant (p_{adj}=0.017) and Δ3 core mutant (p_{adj}=0.009) are significantly higher. No such increased binding was observed for hnRNP A1 that represented a splicing factor not increasing exon inclusion in the *RAD50* minigene (Fig. 1F). Statistical test was performed as ANOVA corrected for multiple comparisons using the Dunnet´s Test with GraphPad, and $p<0.05$ (2 df) was considered nominally significant (n=3). Differences are indicated as *($p<0.05$) or ns ($p>0.05$).

Supplemental Figure 4: Functional complementation of RAD50-deficient fibroblasts

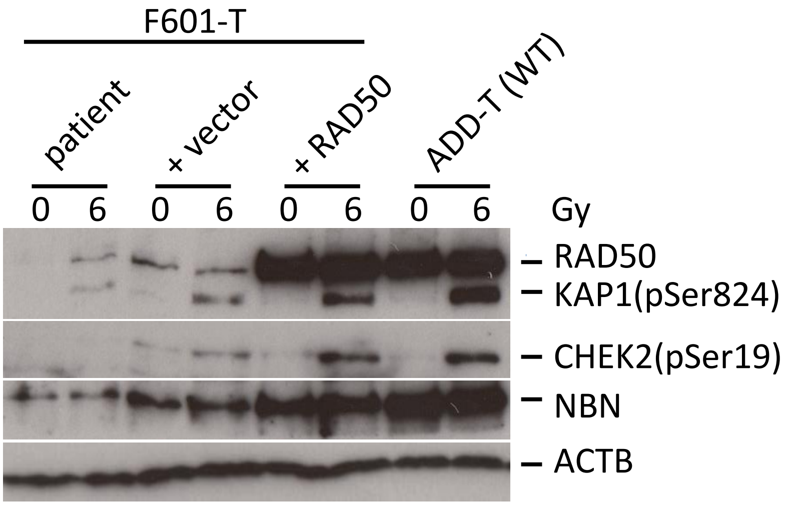

Legend to Supplemental Figure 4: Functional complementation of RAD50-deficient fibroblasts

Immunoblot analysis of protein lysates from large-T immortalized patient fibroblasts (F601-T) and their derivatives expressing either wild-type RAD50 (+RAD50) or vector alone (+vector), as described in the Methods section. Protein levels of RAD50 and NBN as well as phosphorylation of KAP1(Ser824) and CHEK2(Ser19), respectively, were analyzed with specific antibodies after 6 Gy irradiation in comparison to unirradiated cells. RAD50 level was fully restored in the complemented fibroblasts when compared to ADD-T control fibroblasts, derived from a healthy donor. In parallel, NBN levels increased, and KAP1(Ser824) and CHEK2(Ser19) phosphoproteins were detected at increased intensity after 6 Gy irradiation, consistent with improved ATM kinase activity.
