# Supplementary material for: A deep intronic mutation causes RAD50 deficiency through an unusual mechanism of distant exon activation
Source: J Clin Invest. 2024 Dec 12;135(3):e178528. doi: 10.1172/JCI178528 (PMC11785915; doi:10.1172/JCI178528)

A deep intronic mutation causes RAD50 deficiency through an unusual mechanism of distant exon activation

Bousset K. et al.,

- Full unedited blots/gels -

Full unedited gel for Figure 1A:
patient RNA
cDNA analysis

2= patient, 3= control

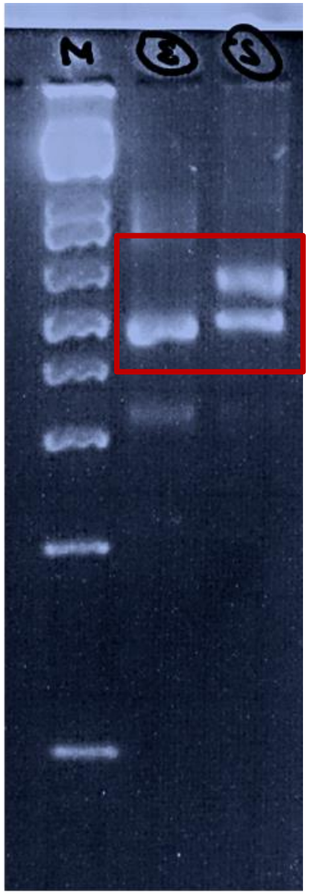

Full unedited blot for Figure 1B:
Western blotting patien cells

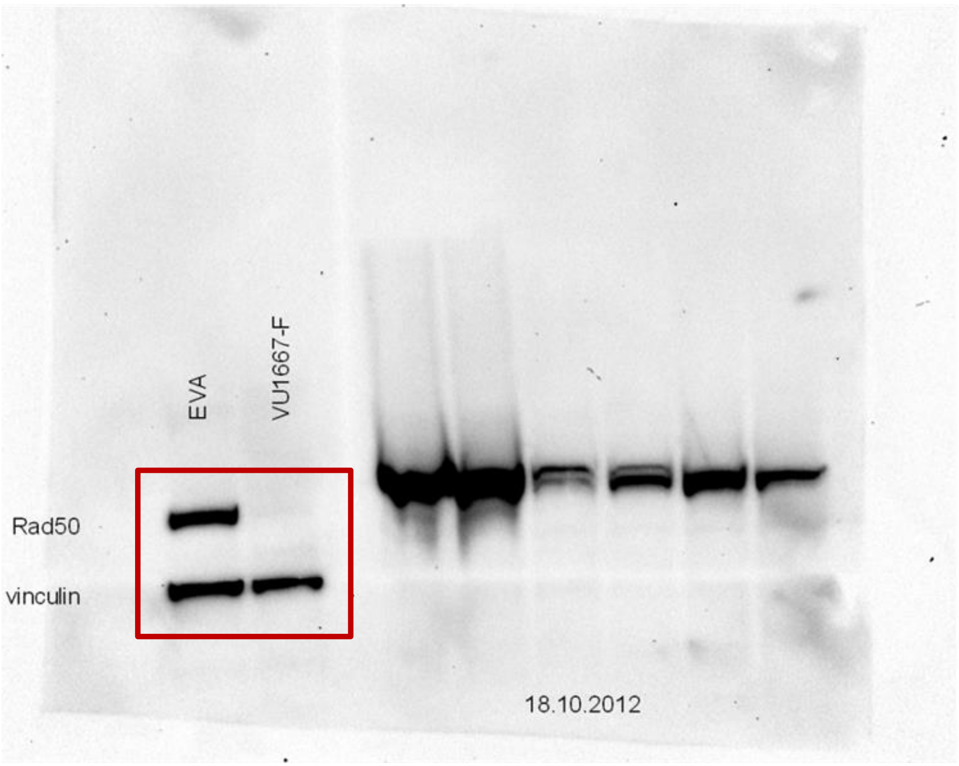

Full unedited gel for Figure 1B: RAD50 minigene (MG), agarose gel, PCR on cDNA

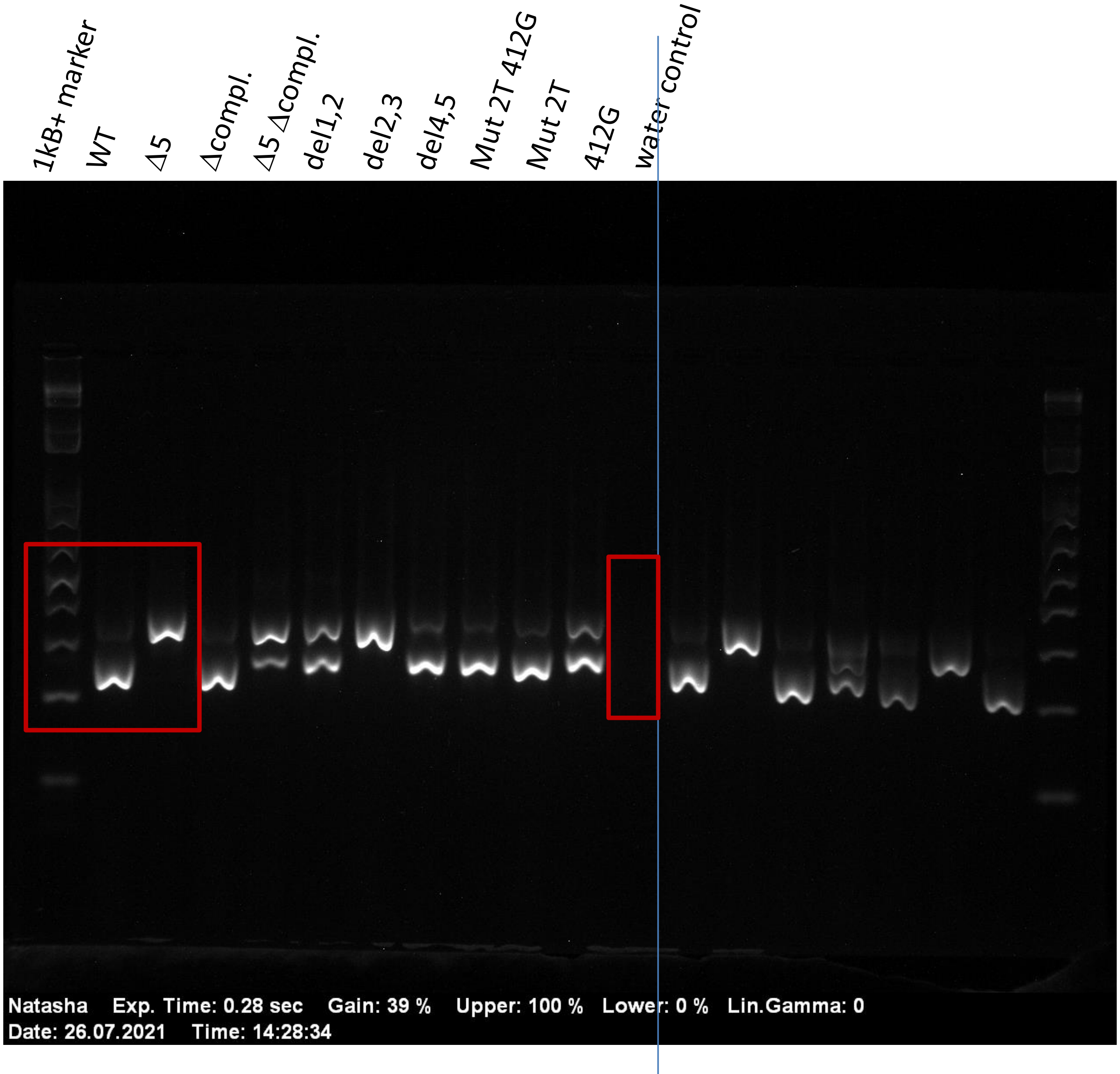

Full unedited gel for Figure 1C: MG; agarose gel, PCR on cDNA

Double dels

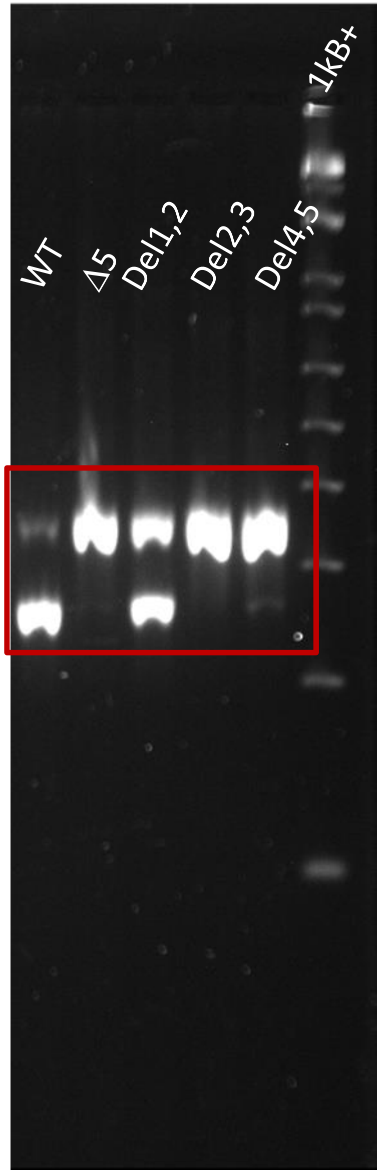

Single dels

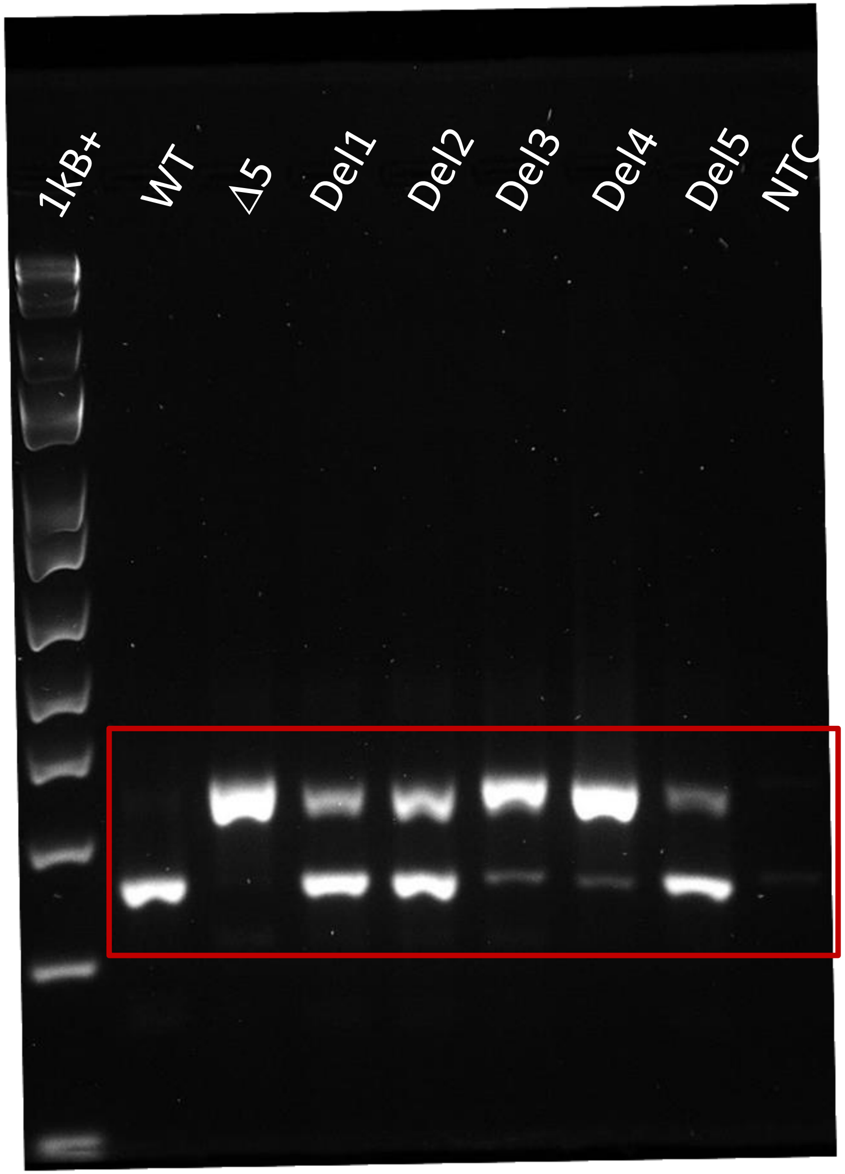

Full unedited gel for Figure 1C: continued

Single nucleotide substitutions MG; agarose gel, PCR on cDNA

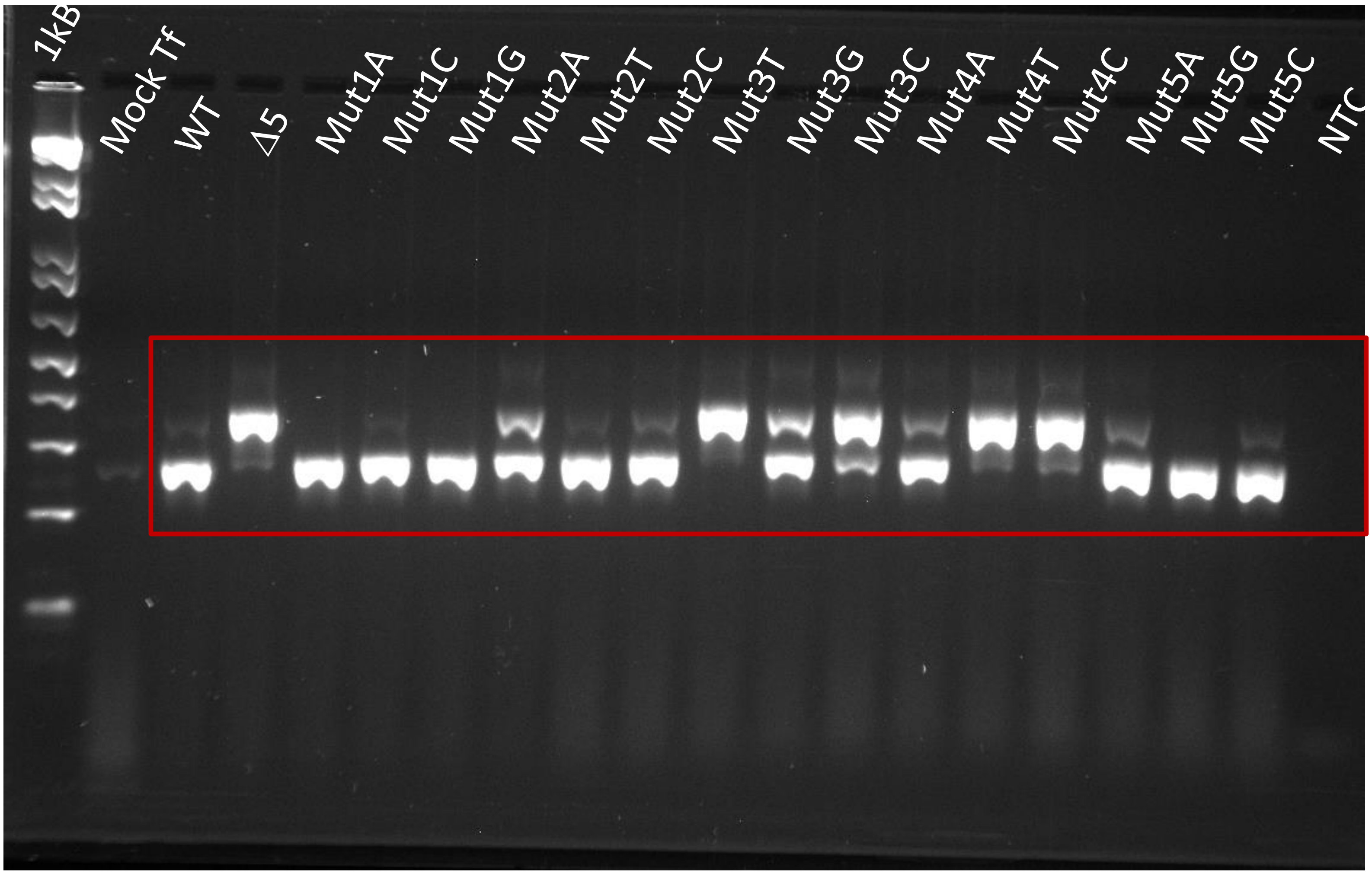

Full unedited gels for Figure 1E: PPT muation MG; agarose gel, PCR on cDNA

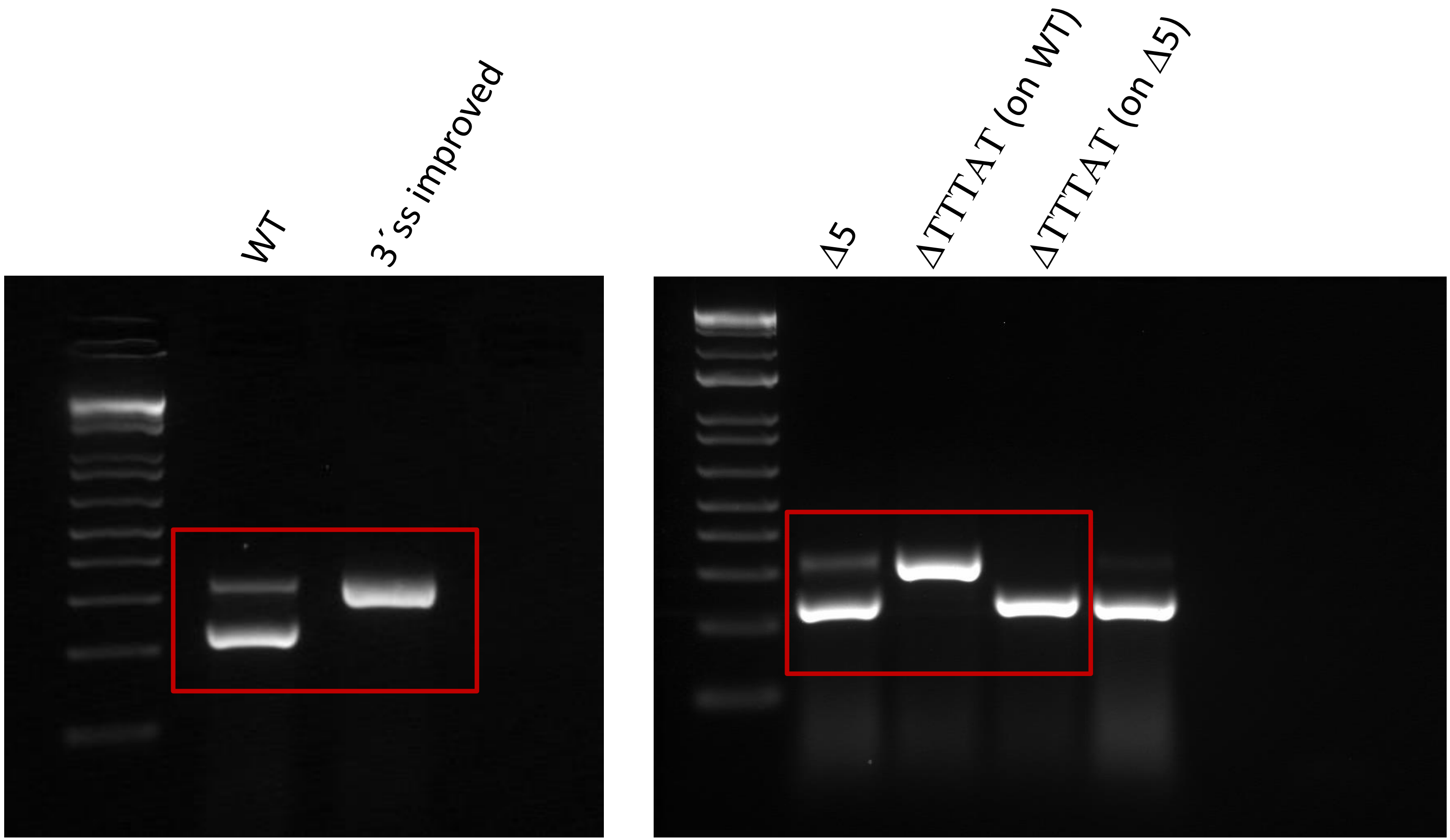

Full unedited gel for Figure 1F: splice factor titration, MG, agarose gel PCR on cDNA

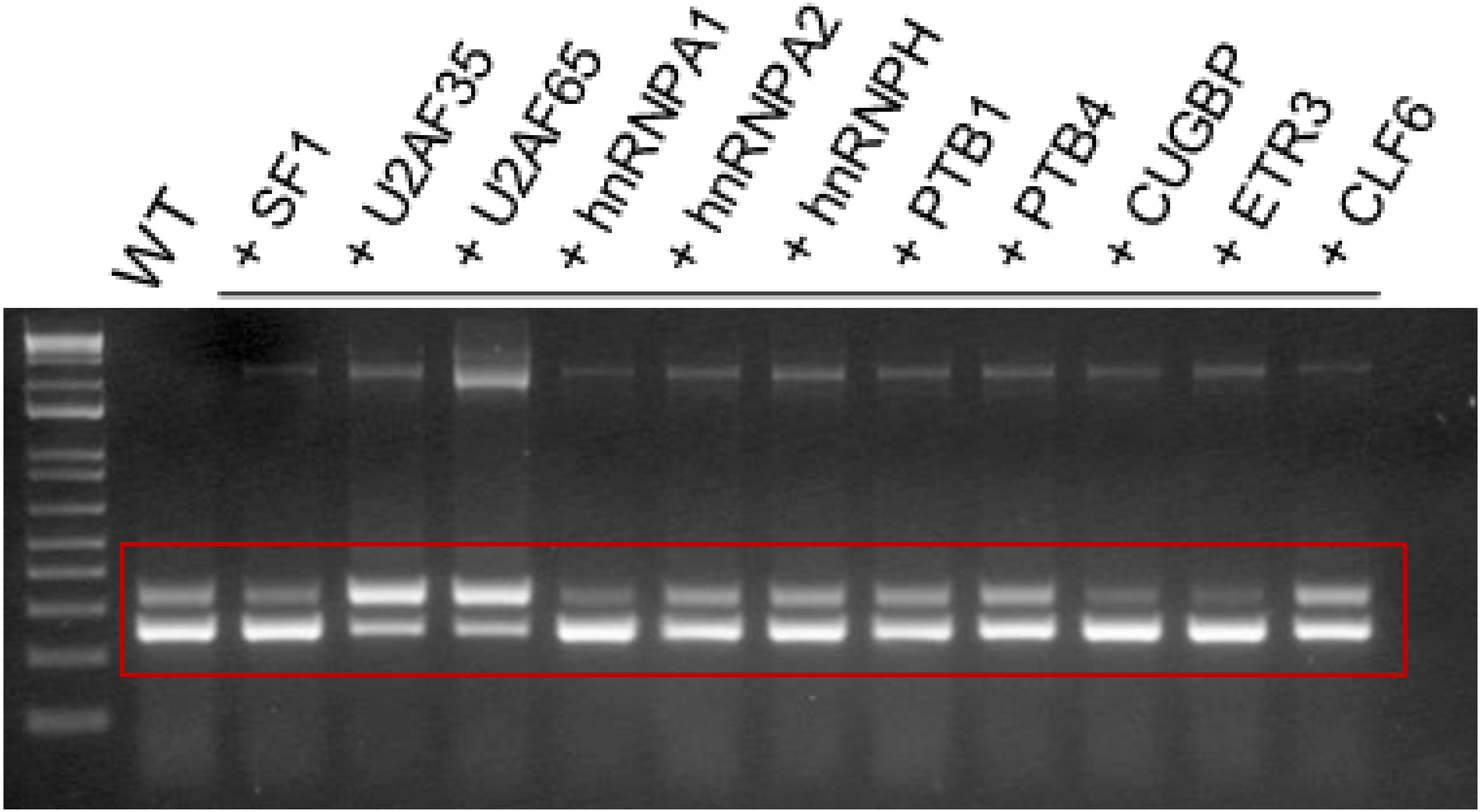

Full unedited blot for Figure 1G: pull down western, upper panel

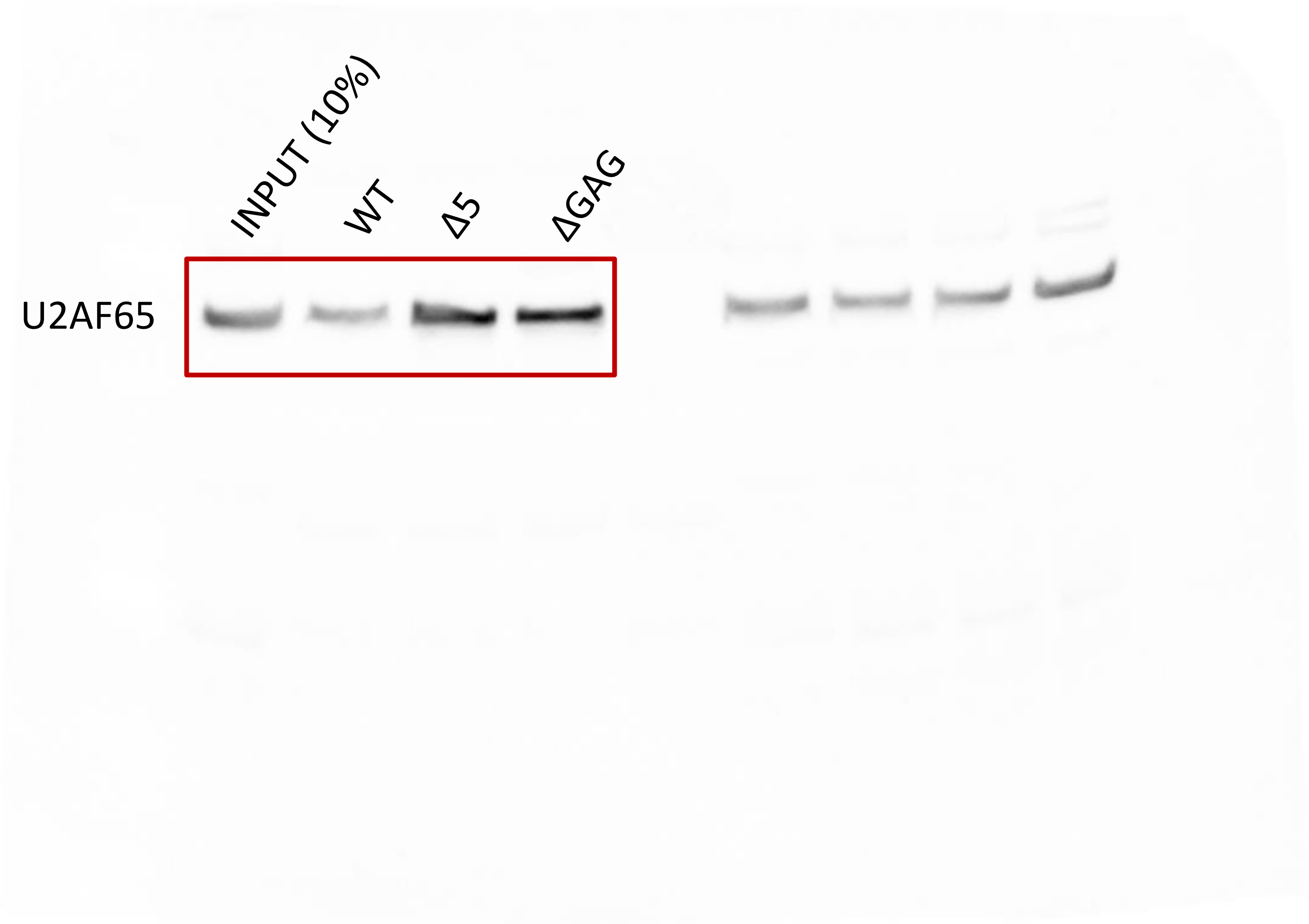

Full unedited blot for Figure 1G: pull down western, midddle panel

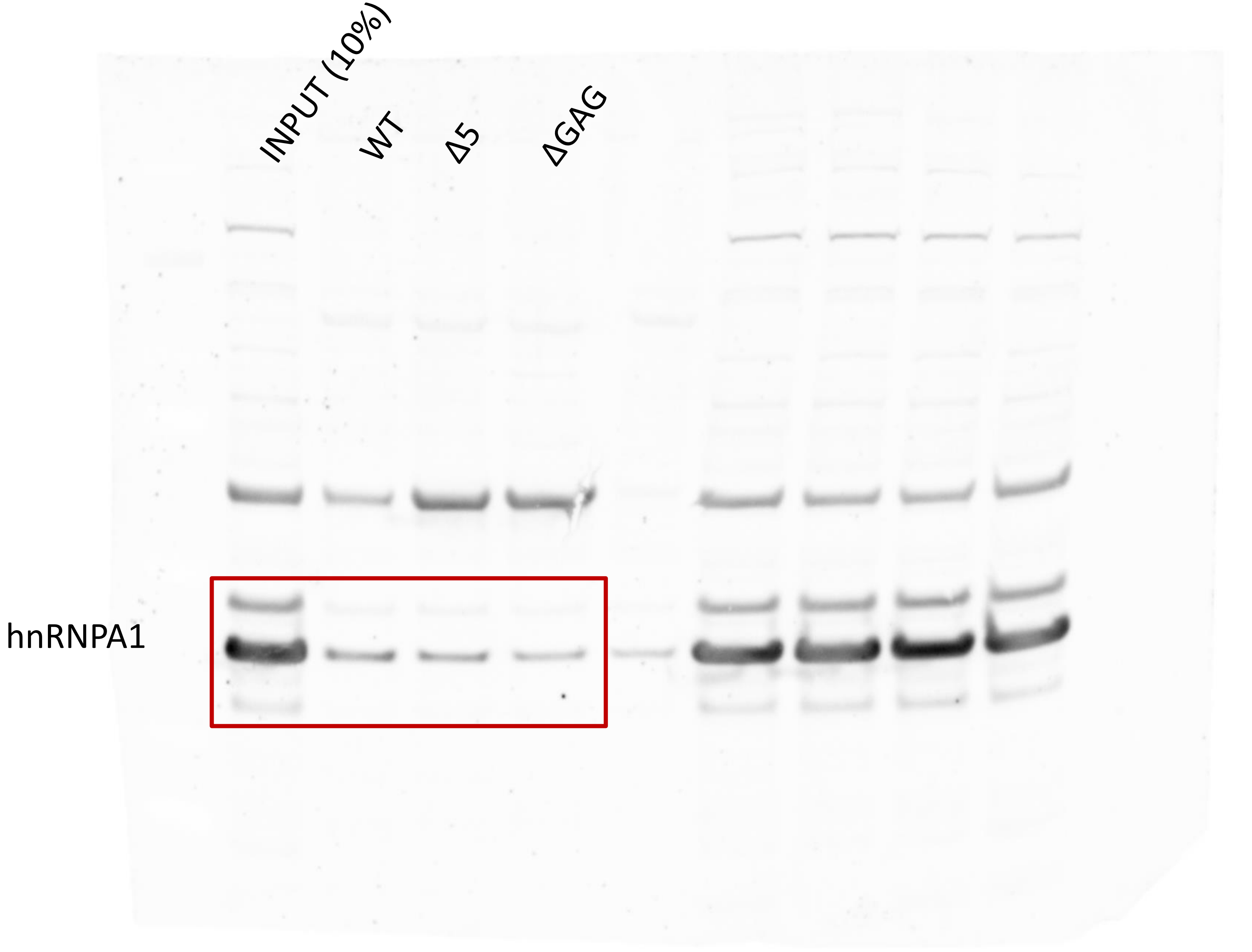

Full unedited blot for Figure 1G: pull down western, lower panel

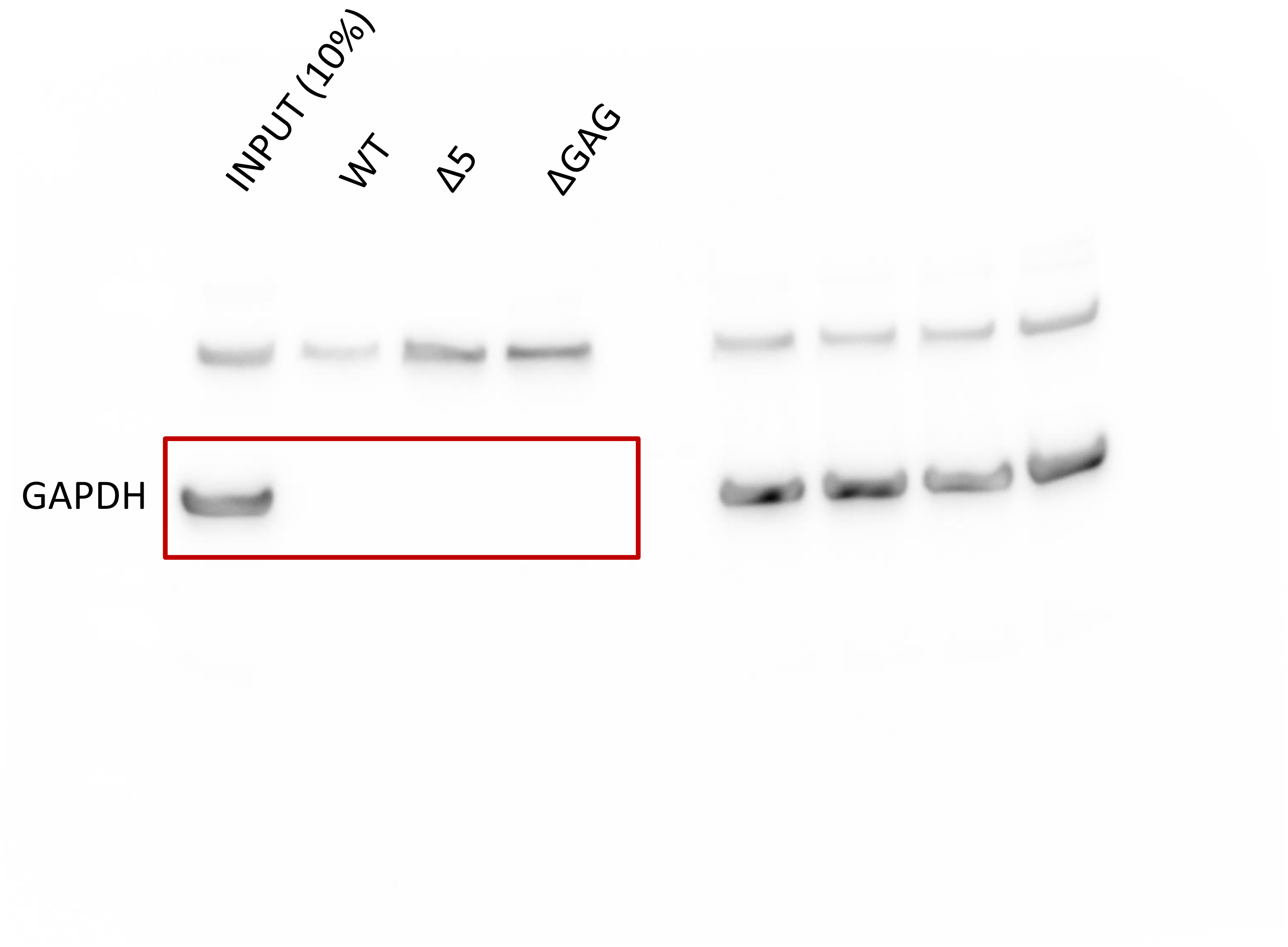

Full unedited gel for Figure 1I: IR AMO, agarose gel (PCR on cDNA)

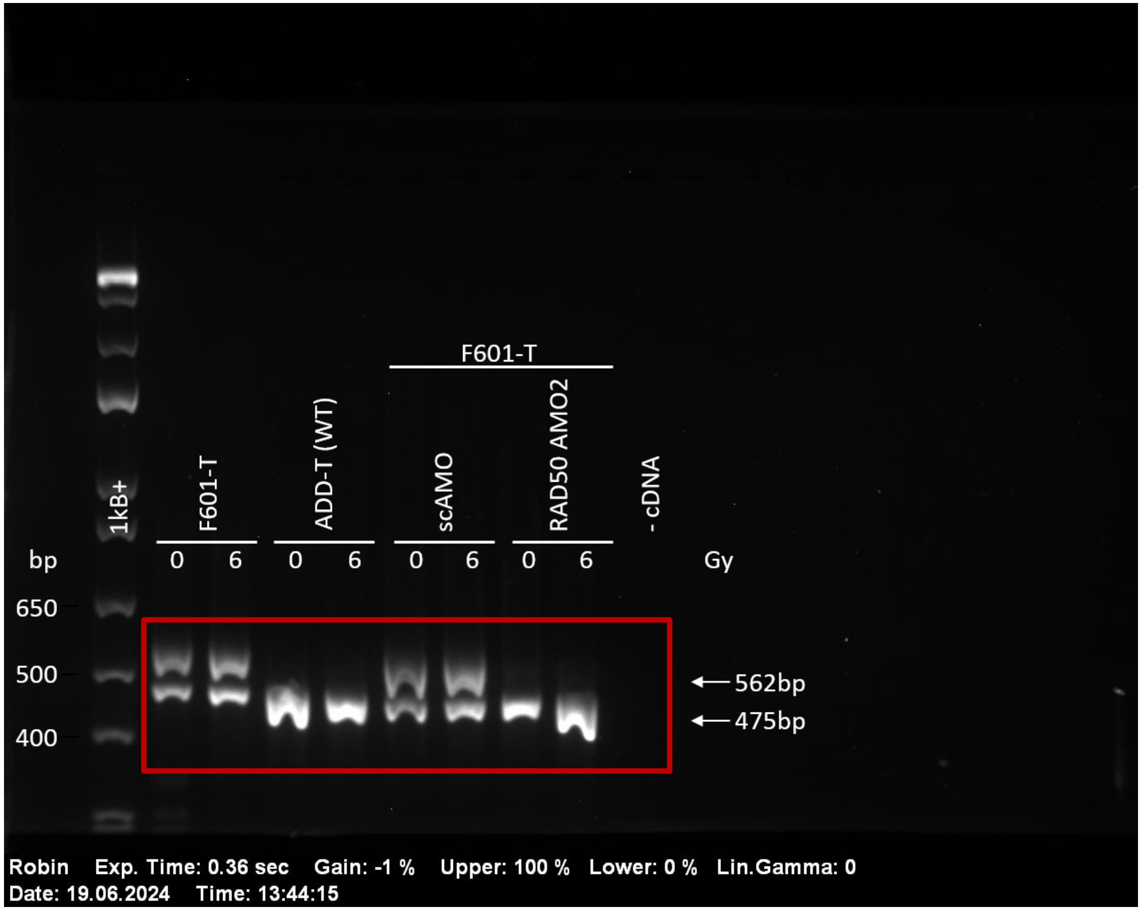

Full unedited blots for 1I: IR AMO, western blots partA

RAD50 & β-actin (cut membrane)

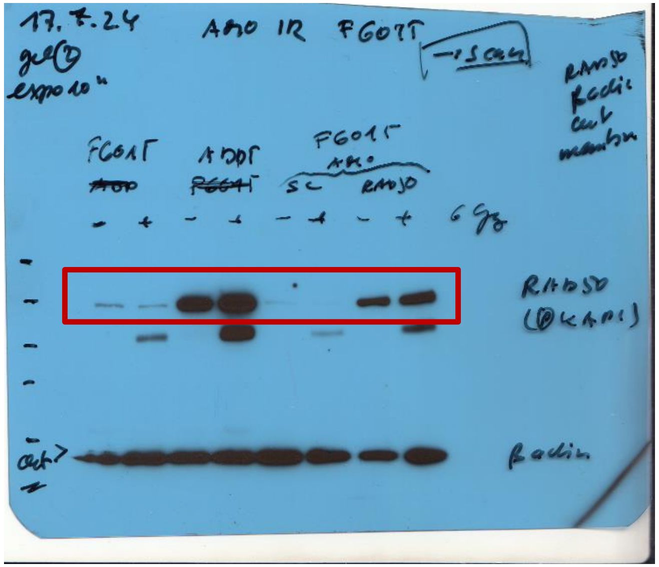

higher exposure

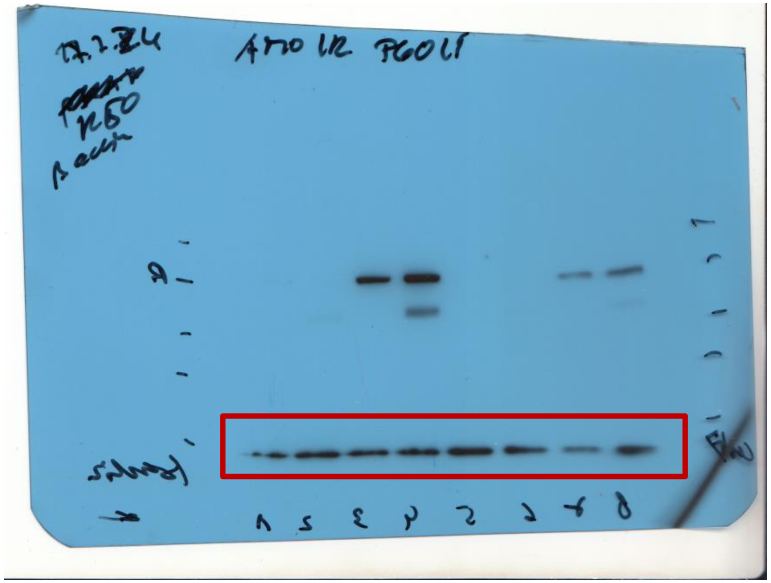

lower exposure

P-KAP (S824)

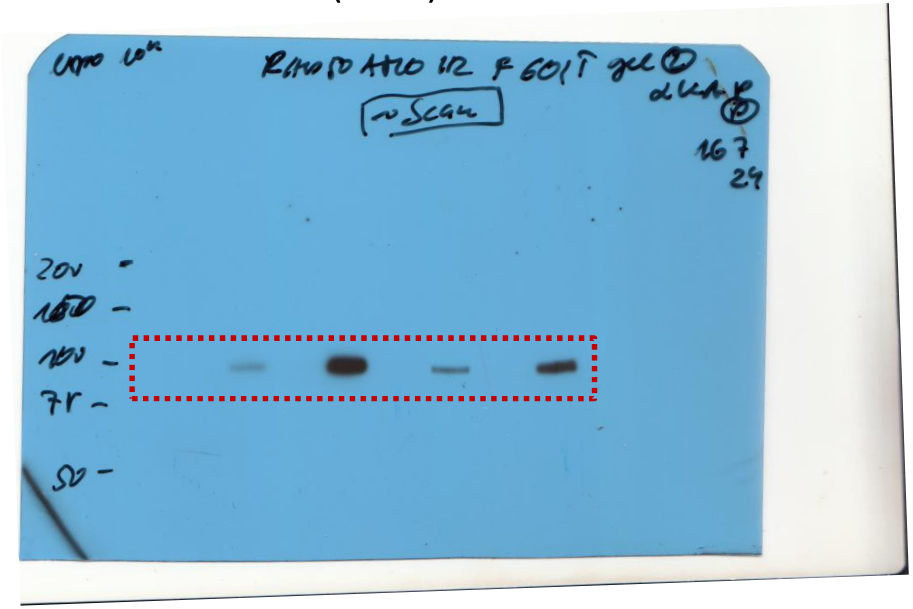

Full unedited blots for Figure 1I: IR AMO, western blots partB

NBN (cut membrane)

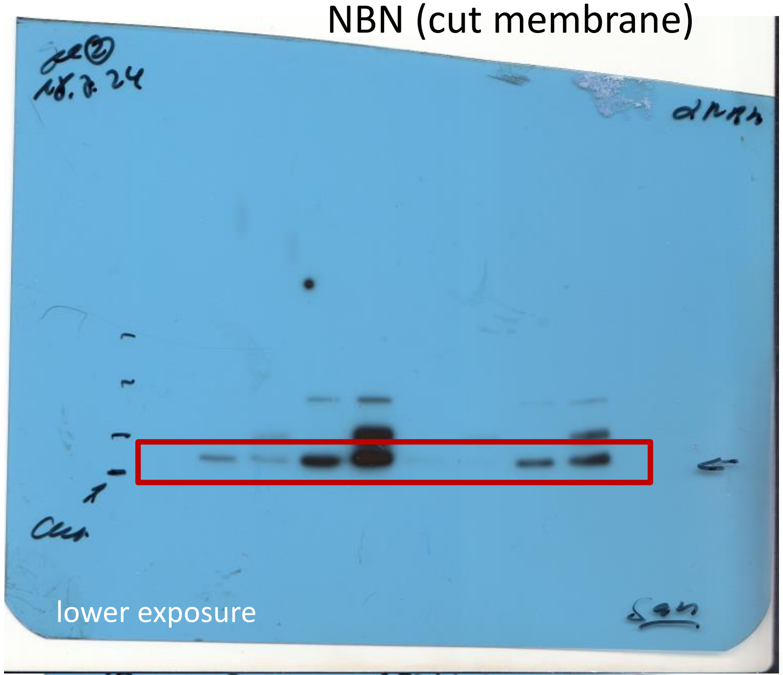

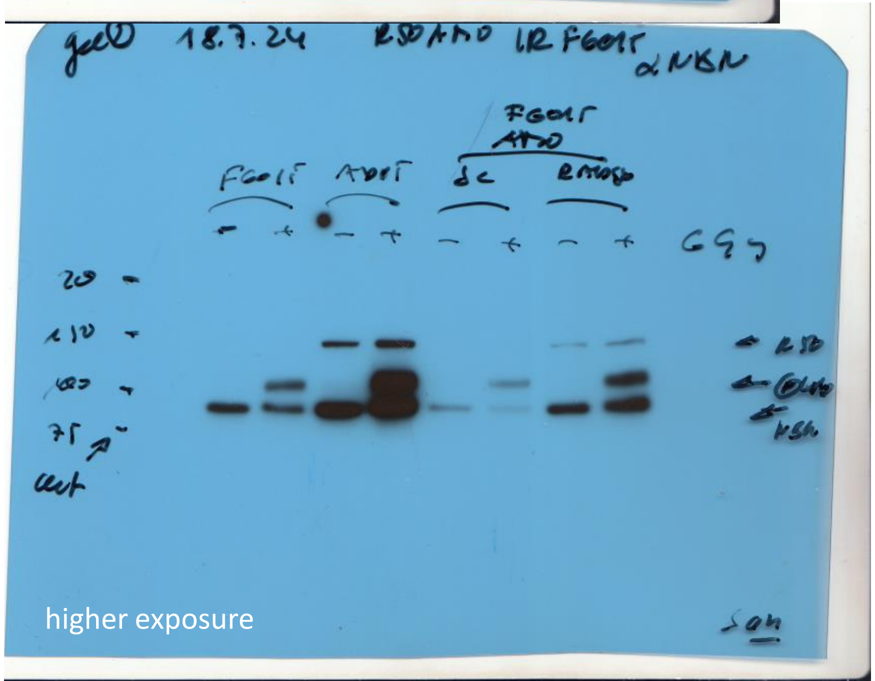

P-CHEK2 (S-19) (cut membrane)

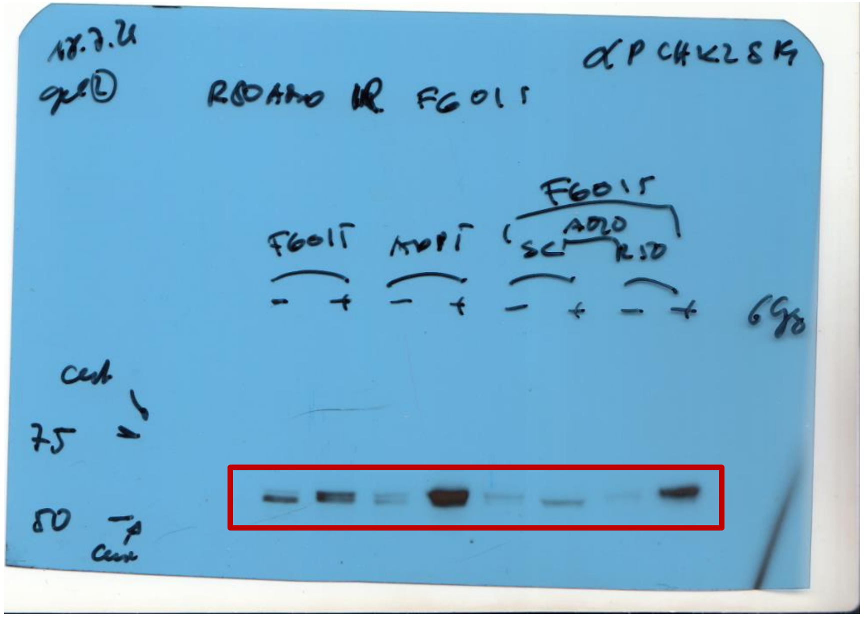

Full unedited gel for Suppl. Figure 2A

Transposition of RAD50 3´splice region to unrelated exon

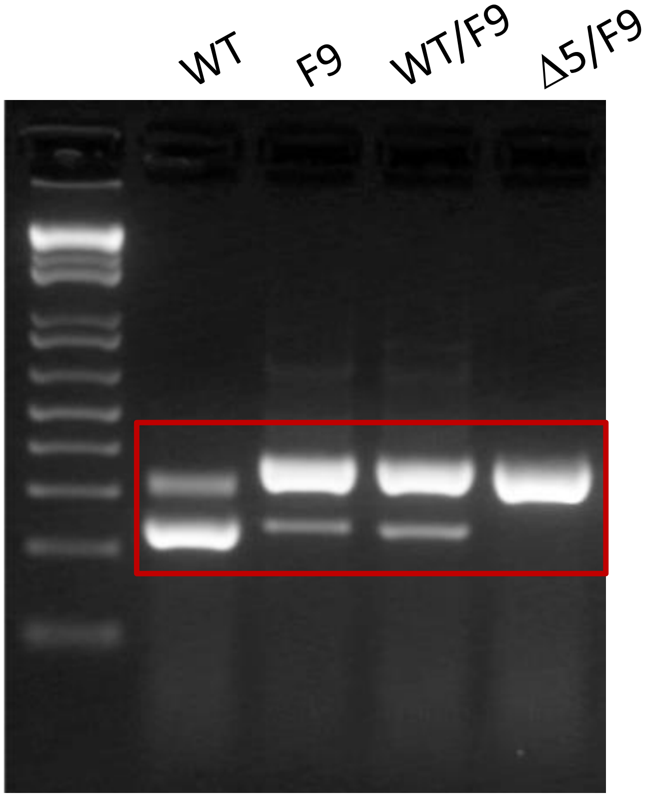

Full unedited blot for Suppl. Figure 2F: Distinct deletions in the Δ5 sequence

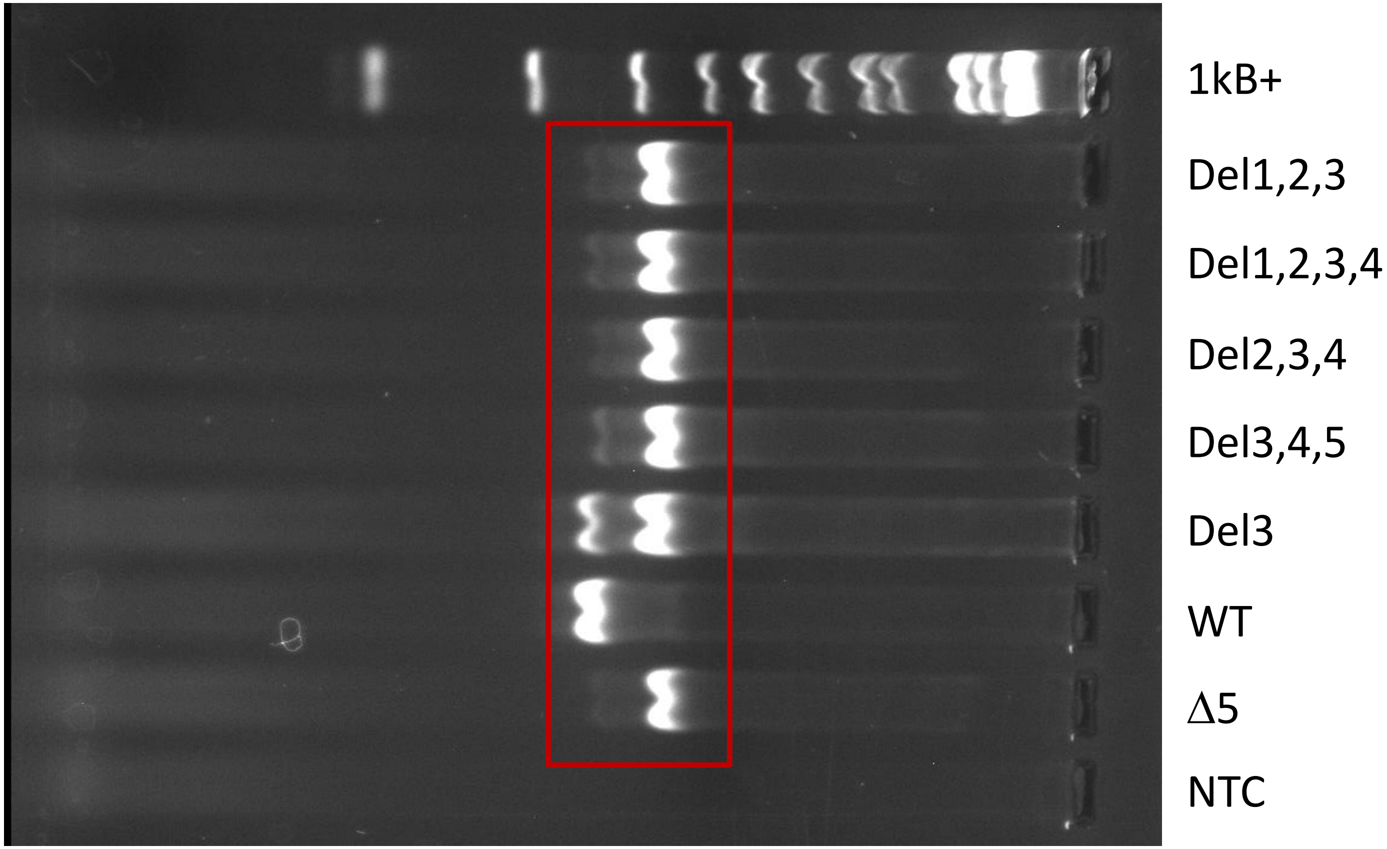

Full unedited blots for SUPPL Figure 4: : Functional complementation of RAD50-deficient fibroblasts partA

RAD50
P-KAP1 S-824

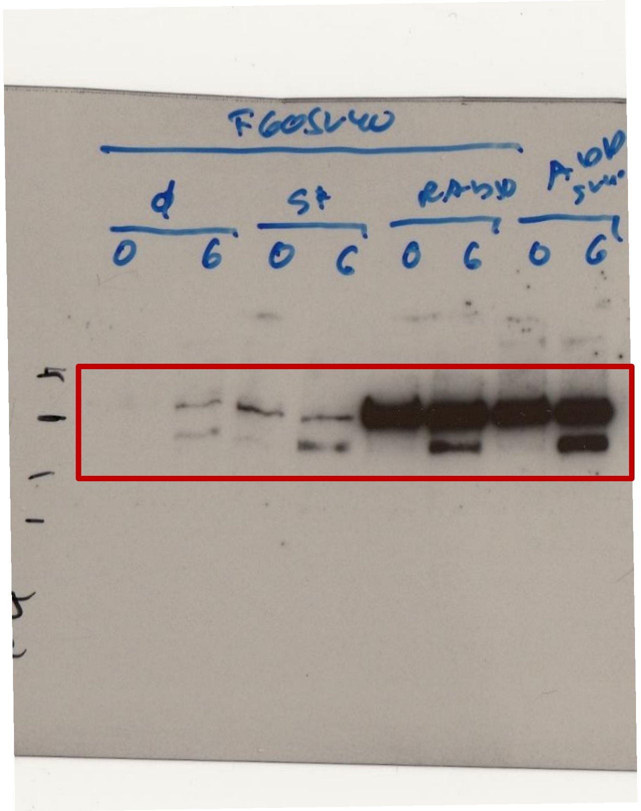

RAD50
P-KAP1 S-824

P-CHK2 S-19

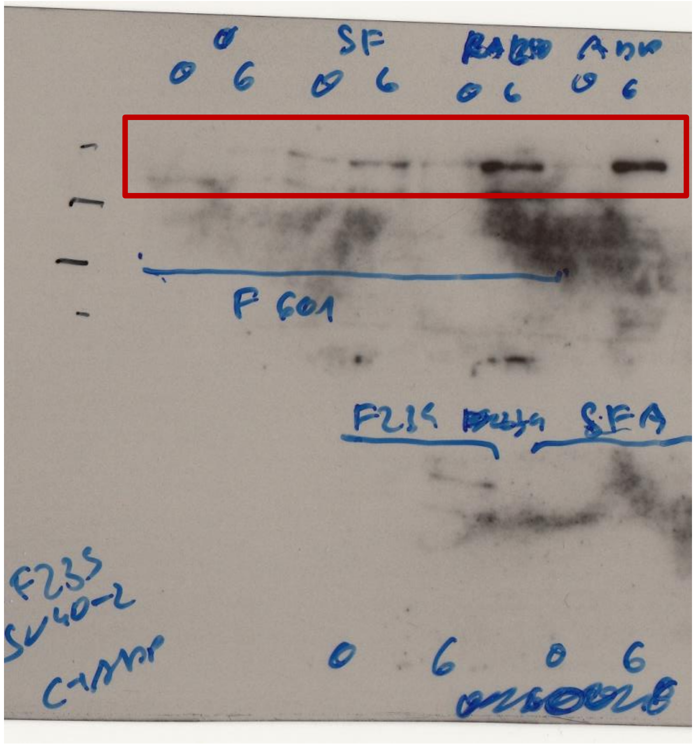

P-CHK2 S-19

Full unedited blots for SUPPL Figure 4: : Functional complementation of RAD50-deficient fibroblasts partB

NBN

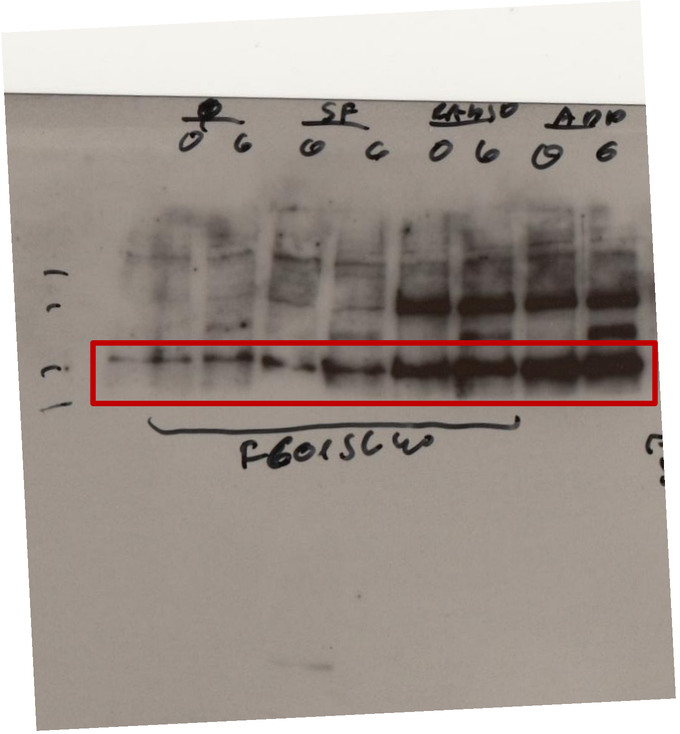

RAD50
P-KAP1 S-824
NBN

ACTB

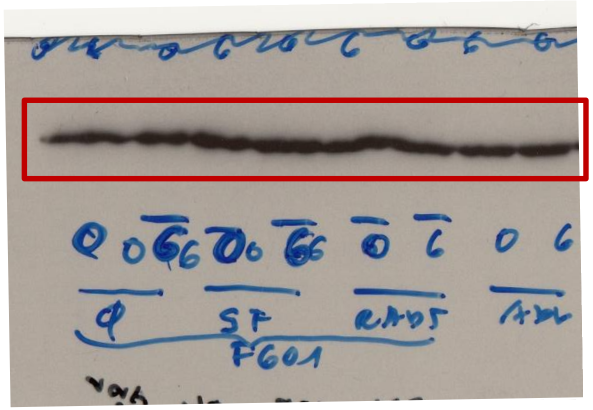

Supplement: Unedited blot and gel images [file jci-135-178528-s118.pdf]
